# Interplay Between Chemotherapy-Activated Cancer Associated Fibroblasts and Cancer Initiating Cells Expressing CD44v6 Promotes Colon Cancer Resistance

**DOI:** 10.3389/fonc.2022.906415

**Published:** 2022-08-02

**Authors:** Shibnath Ghatak, Vincent C. Hascall, Nikos Karamanos, Roger R. Markwald, Suniti Misra

**Affiliations:** ^1^ Department of Regenerative Medicine and Cell Biology, Medical University of South Carolina, Charleston, SC, United States; ^2^ Department Natural Sciences, Trident Technical College, North Charleston, SC, United States; ^3^ Department of Biomedical Engineering/ND20, Cleveland Clinic, Cleveland, OH, United States; ^4^ Department of Chemistry, University of Patras, Matrix Pathobiology Research Group, Patras, Greece

**Keywords:** colorectal cancer (CRC), cancer initiating cells (CICs), cancer associate fibroblasts (CAFs), CD44v6, periostin, IL17A, WNT3A, CD44v6-therapy

## Abstract

Cancer-initiating cells (CICs) drive colorectal tumor growth by their supportive niches where CICs interact with multiple cell types within the microenvironment, including cancer-associated fibroblasts (CAFs). We investigated the interplay between the CICs and the clinically relevant chemotherapeutic FOLFOX that creates the persistent tumorigenic properties of colorectal CICs, and stimulates the microenvironmental factors derived from the CAFs. We found that the CICs expressing an immunophenotype (CD44v6[+]) promote FOLFOX-resistance and that the CIC-immunophenotype was enhanced by factors secreted by CAFs after FOLFOX treatment These secreted factors included periostin, IL17A and WNT3A, which induced CD44v6 expression by activating WNT3A/β-catenin signaling. Blocking the interaction between CICs with any of these CAF-derived factors through tissue-specific conditional silencing of CD44v6 significantly reduced colorectal tumorigenic potential. To achieve this, we generated two unique vectors (floxed-pSico-CD44v6 shRNA plus *Fabpl-Cre*) that were encapsulated into transferrin coated PEG-PEI/(nanoparticles), which when introduced *in vivo* reduced tumor growth more effectively than using CD44v6-blocking antibodies. Notably, this tissue-specific conditional silencing of CD44v6 resulted in long lasting effects on self-renewal and tumor growth associated with a positive feedback loop linking WNT3A signaling and alternative-splicing of CD44. These findings have crucial clinical implications suggesting that therapeutic approaches for modulating tumor growth that currently focus on cell-autonomous mechanisms may be too limited and need to be broadened to include mechanisms that recognize the interplay between the stromal factors and the subsequent CIC-immunophenotype enrichment. Thus, more specific therapeutic approaches may be required to block a chemotherapy induced remodeling of a microenvironment that acts as a paracrine regulator to enrich CD44v6 (+) in colorectal CICs

## Introduction

Human colorectal cancer is a widely studied human tumor type for which the steps from small adenomas to metastatic colorectal cancer (CRC) are clearly defined. In the face of recent progress in cancer therapy and increased knowledge of tumor biology, CRC ranked second in women after breast cancer and third in men after prostate and lung cancers (1). CRC-associated death is primarily caused by cancer recurrence and metastasis. CRC recurrence is defined as local, regional, and distant metastatic recurrence after a disease-free period (2). Moreover, five-year survival in CRC ranges from 90% in early localized stages to less than 10% in advanced, metastatic cases (3, 4).

The vast majority of tumors (90%) are linked to somatic mutations and environmental factors, whereas only a minority of all cancers are caused by germline mutations. Recent studies provide evidence that tumors exist as complex tissues composed of heterogeneous, aberrant cell types containing a hierarchy of cells that differ in morphology, gene expression, proliferative capacity, and invasiveness (5). A small subset of cancer cells known as cancer initiating cells (CICs) display cellular hierarchies from which tumor clones originate with tumor initiation ability, self-renewal capacity, long-term repopulation potential, cell death evasion capacity, and demonstration of extensive proliferating capacity to differentiate into multi cell types (6–11). CICs do not simply survive in seclusion, but rather live in a tumor microenvironment known as “niche”. The niche is the cellular environment in which the CICs exist and interact within the extracellular matrix (ECM) with mesenchymal fibroblast cells called cancer associated fibroblasts (CAFs) (12–15). Additionally, immune cells (16), endothelial cells (17), and paracrine signaling molecules such as growth factors (18), matricellular proteins (19) and cytokines (20) are found in the microenvironment of these niches (21, 22). We hypothesize that paracrine factors secreted by CAFs in the CIC niche interact with specific isoforms (variants) of CD44 receptors on CICs to govern tumor growth.

CD44 is a multi-structural and multi-functional transmembrane glycoprotein that is encoded by a single gene containing 20 exons, ten of which are alternatively spliced to generate the numerous CD44 splice variants (CD44v) (23, 24). The standard isoform of CD44 (CD44s) has no variant exons, is small, and is nearly ubiquitous in vertebrate cells (25). Even though CD44s, CD44v6 and CD44v4-10, are detected in the human gut epithelium (26), experiments using knock-in mice that express either CD44v4-10 or CD44s isoforms have demonstrated that CD44v isoforms, but not the CD44s isoform, promote adenoma formation in Apc/Min/+ mice (26). CD44v6 acts as a coreceptor for many growth factors and cytokines produced by cells in the tumor microenvironment, including HGF (27), VEGF (28), EGF (29) and TGFβ1 (30, 31). We and others have shown that CD44 receptors can serve as a signaling platform that integrates cellular microenvironmental cues with growth factor and cytokine induced receptor tyrosine kinase or non-tyrosine kinase signals and transduces these signals to membrane-associated cytoskeletal proteins (27, 28, 32–36). This regulates a variety of genes related to apoptosis resistance including MDR1 (37–40), suggesting a mechanism that could explain CIC’s chemoresistance.

While cytokines and chemokines within the tumor environment are well known paracrine and autocrine signaling factors, in this study, we have focused on stromal proteins secreted by CAFs whose roles are less understood in the context of the tumor microenvironment associated with CD44v6(+)-CICs. One of the stromal factors is periostin (PN), a matricellular protein (41). It is frequently upregulated in various types of human cancers, including CRC (41–45). PN is observed mainly in tumor stroma and at a minor level in the cytoplasm of cancer cells, and stromal PN has a key role in regulating CIC maintenance and expansion during metastatic colonization by increasing WNT signaling (19). Similarly, CAFs create a chemo-resistant niche in CRC by releasing cytokines, including IL17A, a colorectal CIC maintenance factor (20). Likewise, the functional expression of WNT activity defines CRC stem cells and is regulated by the microenvironment (12, 46). WNT-pathway activation results in nuclear accrual of its main effector protein, β-catenin, which interacts with transcriptional regulators including leukocyte enhancer factor-1 (LEF-1) and T cell factor (TCF), which leads to WNT responsive gene expression (47).

Although much work has been done on molecular structure and tumorigenic functions of CD44v6, most of these functions are contributed by tumor cells instead of CICs. Thus, in this study, we present evidence for the role of CD44v6(+) expressed by CICs in communicating within the tumor microenvironment and in regulating CIC stemness properties to sustain drug resistance, which has not been clearly understood. We showed that FOLFOX therapy induces reorganization of the tumor micro-environment that supports a cellular hierarchy in CRC, enriching the undifferentiated highly tumorigenic CD44v6(+)CIC subpopulation through secreted factors derived from CAFs. We found that human colorectal tumor tissue contains cancer initiating cells defined by CD44v6 expression that are exclusively tumorigenic and highly resistant to standard chemotherapy, and the CIC tumorigenic potential is stimulated significantly. Our data also suggest that combinations of 5-fluorouracil [5-FU], oxaliplatin [OXA], and leucovorin induce reorganization of the tumor microenvironment that supports a cellular hierarchy in CRC, enriching the undifferentiated highly tumorigenic CD44v6(+)CIC subpopulation through secreted factors derived from CAFs. FOLFOX stimulation increased the ability of CAFs to stimulate a relative proportion of fibroblasts to the epithelial component (α SMA versus EpCAM). Subsequently CAFs create a chemo-resistant niche by releasing pre-dominantly PN, WNT3A and IL17A. Exogenous addition of either PN, IL17A or WNT3A increased CIC tumorigenic function and maintenance. Especially, these factors were overexpressed by colorectal CAFs in response to FOLFOX with expression validated directly in patient-derived specimens. Periostin and IL17A sustain a WNT3A induced maintenance of CD44v6(+) CICs. Additionally, our data also revealed that the tumorigenic potential of these CICs together with the CAFs subpopulation significantly increased in secondary and tertiary subcutaneous xenograft tumors. In contrast, tumorigenic potential of Non-CICs plus CAFs was completely lost in secondary, tertiary and quaternary subcutaneous xenograft tumors suggesting that non-CICs are differentiated non-tumorigenic cells. These results provide evidence that drug resistance and long-term tumorigenic potential are restricted to the CD44v6 expressing CIC population, and chemotherapy induces remodeling of the tumor microenvironment to support the tumor cellular hierarchy through secreted factors. These results have central clinical significances as most chemotherapeutic methods focus on blocking cell-autonomous mechanisms without reflecting on the crosstalk between CICs and CAFs that may promote the specific CD44v6-signaling required for maintenance of CIC resistance and tumor recurrence through sustained WNT3A/β-catenin/TCF4 signaling.

## Materials and Methods

### Institutional Review Board (IRB) Statement

The Medical University of South Carolina (MUSC) IRB determined that this research project meets the criteria for “Not Human Subjects”.

### Institutional Animal Care and Use Committee (IACUC) Statement

All animal studies described were approved by the Medical University of South Carolina (MUSC) IACUC (protocol # IACUC – 2019-00829; Approval period 08/03/2020- 09/24/20221) and conducted in accordance with the National Institutes of Health Guide for the Care and Use of Animals.

### Materials

Dulbecco’s Modified Eagle’s Medium (DMEM), Eagle’s Minimum Essential Medium (EMEM), McCoy’s 5A Medium, F-12K Medium, Leibovitz’s L-15 Medium, L- Glutamine, Sodium pyruvate, Penicillin (100 µg/ml) and Streptomycin (100 µg/ml), sodium pyruvate, 0.05% EDTA solution (Versene), Phosphate buffered saline (PBS, Calcium and Magnesium free), and 0.05% Trypsin were from Corning Inc. Fetal Bovine Serum (FBS) was from Atlanta Biologicals. Amphotericin B- Hyclone was from *Thermo Fisher Scientific*, Waltham, MA, USA. Nonidet P-40, EGTA, sodium orthovanadate, glycerol, phenylmethylsulphonyl fluoride, leupeptin, pepstatin A, aprotinin and HEPES were from Sigma-Aldrich, Inc. St. Louis, MO, USA. Recombinant human WNT3A protein (5036-WN) periostin protein (CF 3548-F2), IL-17A protein (CF 317-ILB) were from R&D Systems, Inc. Minneapolis, MN, USA. The anti-Active-β-catenin antibody (05-665, anti-ABC antibody clone 8E7)from Millipore Sigma, Burlington, MA, USA, and the anti- β-catenin antibody (610153, mouse IgG1, BD, Tempe, Arizona, USA) were used for total β-catenin detection in western blotting analysis. The antibodies p-LRP6 (Serine 1490) (#2568, Rabbit IgG), LRP6 (#2560, Rabbit mAb clone C5C7), TCF4 antibody (#2569, Rabbit mAb clone C48H11) were from Cell Signaling Technology, Inc. Danvers, MA, USA. P-Glycoprotein (MDR1) western blotting antibody (PA5-28801, Rabbit Polyclonal against Human) was from Invitrogen-*Thermo Fisher Scientific*, Waltham, MA, USA. β β−Tubulin Antibody (D-10) (sc-5274, Mouse monoclonal IgG_2b_ κ, SCBT), Mouse anti-rabbit IgG-HRP (sc-2357, IgG, SCBT), Rabbit anti mouse IgG-HRP (sc-358914, IgG, SCBT), and Western blotting Luminol reagent (sc-2048, SCBT) were purchased from Santa Cruz Biotechnology Inc., Dallas, Texas, USA. Blocking antibodies for CD44v6 (BBA13, Monoclonal Mouse IgG_1_ Clone # 2F10, R&D), and isotype control (MAB002, IgG1, R&D) were from R&D Systems, Inc. Minneapolis, MN USA. The mouse IgG1 antibodies were from R&D Systems, Inc. Minneapolis, MN, USA. Blocking antibody for protein WNT3A (703666) was from R&D Systems, Inc. Minneapolis, MN USA. Rabbit monoclonal IgG clone 1H12L14) was from *Thermo Fisher Scientific.* Waltham, MA, USA. Blocking antibody for periostin (OC-20) was from Adipogen Corporation, San Diego, CA, USA. Blocking antibody for IL17A Monoclonal (eBioMM17F3), and the mouse IgG1 antibodies were from R&D Systems, Inc. Minneapolis, MN USA. pcDNA3-WNT3A-V5 was a gift from Marian Waterman (Addgene plasmid # 35927; http://n2t.net/addgene:35927; RRID : Addgene #3 5927). Periostin cDNA was a gift from Dr. Akira Kudo, School of Dentistry, Showa University, Tokyo, Japan.

### Cell Line

SW480(CCL-228) was maintained in Leibovitz’s L-15 medium that was purchased from ATCC (Manassas, Virginia, USA) in a humidified atmosphere in the presence of 10% FBS, Penicillin (100 µg/ml) and Streptomycin (100 µg/ml) in 5% CO_2_ at 37° C.

### Generation of FOLFOX Resistant Cells

To determine the mechanisms of FOLFOX resistance, we selected SW480 cells out of 7 cell lines (as shown in in our companion paper (48), which have low basal levels of CD44v6 gene expression. Using this cell line, we determined the IC_50_ values for 5-Flourouracil (5-FU) and for oxaliplatin (OXA) (as shown in in our companion paper (48), because these molecules are the components of FOLFOX. To determine these IC_50_ values, cells were separately pretreated with various concentrations of 5-FU, or OXA, or vehicle. After a 24-h incubation at 37° C, growth assays were analyzed as described below. The 50% inhibitory concentration (IC_50_) was identified as a concentration of drug required to achieve a 50% growth inhibition relative to untreated controls. The average IC_50_ value for SW480 cells for 5-FU is 47 µM and for OXA is 9.6 µM. FOLFOX resistance cells were generated by incubating the sensitive parental SW480 cells (SW480-S) with increasing concentrations from 1x FOLFOX (50 µM 5-FU + 10 µM OXA + 1 µM leucovorin) to 5 x FOLFOX over 3 days. This exposure and withdrawal cycle was repeated five times for each dose of FOLFOX. The surviving cells were cultured in normal medium for 5 days. The resistances of these resistant clones were compared to sensitive pairs by determining the numbers of colonies in soft agar growth with 1x FOLFOX - 5x FOLFOX treatments.

### Cell Viability and Apoptosis Assays

Five thousand cells were plated in triplicate into 96-well plates containing appropriate growth media and incubated overnight. After 16 hours growth, cultures were incubated in media containing no serum for 16 hours at 37°C in 5% CO_2_, 95% air. Vehicle or chemotherapy drug was added to the plate. In each experiment, a total of five plates (6 wells/treatment) were used. Experiments were repeated 3 times. The growth of the cells was determined by measuring increases in readings of ATP levels for viability (CellTiter-Glo, Promega). Cell apoptosis was determined by the Caspase-Glo^®^ 3/7 assay (Promega) using DEVD-amino luciferin substrate. The luminescent signal is proportional to caspase 3/7 activity and was measured using a luminometer (Perkin Elmer).

### Tissue Collection and Isolation of CICs

All human drug resistant human CRC tissues were acquired from primary human colorectal tumor patient specimens undergoing colorectal resection, in agreement with human experimental guidelines and the ethical standards of the institutional review board (IRB). Human protocols were approved by the Institutional Review Board of the Medical University of South Carolina. The IRB has determined that this research project meets the criteria for ‘Non Human Subjects’ research. Patient-derived (PD) biopsies collected from 5-FU resistant (PD-5FUR), Oxaliplatin (PD-OXAR), and FOLFOX (PD-FR) tumor specimens and our FOLFOX resistant (FR), Oxaliplatin resistant (OXA-R), 5-flurouracil resistant (5-FUR) cell clones. SW480-S (sensitive) cells were maintained through subcutaneous (SQ) xenografts in the flanks of immunocompromised [NOD-SCID/IL2Rγnull (NSG)] mice and in SCID mice, respectively. Fresh tumors from normal colonic tissue and colorectal PD-FR, PD-OXAR, PD-5FUR, SW480-FR, SW480-OXAR, SW480-5FUR, and SW480-S SQ tumors were rinsed with DMEM (Life Technologies) supplemented with 200 units/mL of penicillin, 200 µg/mL of streptomycin, and 4 units/mL of amphotericin B, followed by incubation with 300 units/ml of collagenase (Worthington Biochemical) at 37° C for 3 hours. A single cell suspension was obtained by filtration through a 40 µm filter. After discarding lymphocytes by gradient centrifugation, the cells were processed for sphere formation (see below for methods), and sphere-propagated cells were subjected to fluorescence-activated cell sorting (FACS) buffer [Phosphate-buffered saline (PBS) + 2% BSA + 1 mM EDTA + 0.1% sodium azide], incubated with Fc blocking reagent (Millenia Biotech), and stained with directly conjugated antibodies by incubating on ice for 20 minutes.

Flow cytometry was done in a cell sorter. To enrich for CICs, single cells were labeled with a phycoerythrin (PE)-conjugated monoclonal antibody against CD44v6 (Miltenyi Biotec), and then analyzed for the expression of Fluorescein-5-isothiocyanate (FITC)-conjugated monoclonal antibody against EpCAM (R&D Systems). Purified CD44v6+/EpCAM+ and CD44v6-/EpCAM+ cells from various tumors were cultured separately and grown in fresh CIC growth medium. CICs were cultured in serum-free media with basic fibroblast growth factor (bFGF, 10 ng/ml; R&D Systems) and epidermal growth factor (EGF, 10 ng/ml; R&D Systems) for 2 weeks. Then, the cultured CD44v6+/EpCAM+ and CD44v6-/EpCAM+ cells were subjected to flow cytometric analysis for isolation of CD44v6+EpCAM+ALDH1+CD133+ (designated as CICs), and CD44v6-EpCAM+ALDH1+CD133+ (designated as Non-CICs) using appropriate fluorescence-conjugated antibodies as described in our companion paper (48). CICs were cultured in serum-free media with basic fibroblast growth factor (bFGF, 10 ng/ml; R&D Systems) and epidermal growth factor (EGF, 10 ng/ml; R&D Systems).

### Tissue Collection and Isolation of CAFs

Single cell suspensions from a dissociated FOLFOX resistant (FR) patient colorectal tumor (PD-FR) were sorted by FACS using PDGFR-*α*-PE and EpCAM-FITC. Percentages of EpCAM(-)/PDGFR-*α*(+) (CAFs) and EpCAM(+)/PDGFR-*α*(+) (Non-CAFs) in total unsorted cell populations were quantified. The integrities of EpCAM(-)/PDGFR*α*(+) gated CAFs from the dissociated CRC cells from the patient colorectal tumors (PD-5FUR, PD-OXAR, PD-FR) and from the sensitive and FOLFOX resistant SW480/SQ tumor tissues were confirmed by QPCR analyses for CAF-associated markers FSP1, FAP, PDGFR-*α*, and epithelial cell marker EpCAM (negative control). EpCAM (-)/PDGFR-*α*(+) cells referred to as ‘CAFs’ were isolated from the indicated tumor tissues. The high PDGFRα positive population was gated in a range of 4–20% (depending on the percentage of the total positive cell population of each sample) of the tail of the positive cells. These EpCAM(-)/PDGFR-*α*(+) cell populations (CAFs) were cultured in DMEM with 10% BSA for 12 days, and were sorted by FACS using *α*-SMA-PE and EpCAM-FITC antibodies. The percentages of the sorted α-SMA(+)/PDGFR-*α*(+) cells in total unsorted CAFs are designated as enrichment of active CAFs. Dead cells were eliminated by using the viability dye DAPI. Isotype controls were used to establish proper gates.

### Tumor Sphere Formation

We followed the tumor sphere formation assay protocol from the Creative Bio-array (Shirley, NY, USA). An optimized serum substitute (1 x B27 supplement) (from Creative Bio array) was freshly added to tumor formation medium (500 ml Dulbecco’s Modified Eagle Medium/F12) containing 20 ng/ml epidermal growth factor, 10 ng/ml basic fibroblast growth factor, 5 μg/ml insulin, and 0.4% bovine serum albumin. After harvesting the cells, 200 live cells/200 µl of tumor sphere medium were suspended on ice and mixed well for plating. PBS was added to the first and last columns (column 1 and 12) of the 96-well plate to help minimize medium evaporation. This leaves 10 wells available for each row. 200 μL aliquots of the cells were suspended in tumor sphere medium and added into each well (200 cells per well). For each treatment, CICs were seeded into the wells of 2 rows for a total of 20 wells. Vehicle or 20 ng/ml of WNT3A, or PN, or IL17A, or a chemotherapeutic drug was added. The upper and lower edges of the 96-well plate were sealed with laboratory tape to avoid evaporation of medium, and cells were placed in an incubator set to 37°C and cultured in 5% CO_2_ for 10 -14 days. These proteins/cytokines were added in optimum media for 24 hours, and this addition was renewed every 3 days. The medium was changed after 48 hours. After stipulated times of incubation, tumor sphere numbers were counted under a phase-contrast microscope using the 40X magnification lens. Data are presented as a percentage of wells containing tumor spheres compared to the total number of wells.

### Cell Lysis, Immunoprecipitation and Immunoblot Analysis

Cells were cultured until they were 75% confluent. They were then washed twice at 4° Cwith phosphate-buffered saline (PBS), and harvested with 0.05% Versene. The cells were pelleted by centrifugation at 5,000 x g for 2 minutes at 4°C. The pellets were treated for 30 minutes with the lysis buffer containing 1% Nonidet P-40, 0.3 M NaCl, 1.5 mM MgCl2, 0.2 mM EDTA, 5 mM sodium orthovanadate, 10% (v/v) glycerol, 100 µg/ml phenylmethylsulphonyl fluoride (PMSF), 1 µg/ml leupeptin, 1 µg/ml pepstatin A, 1 µg/ml aprotinin, and 50 mM HEPES, pH 7.5. For immunoprecipitation, the cell extracts (1 mg total proteins) were precleaned by rotation for 1 h with 20 µl of protein G-Sepharose beads (Santa Cruz, CA). The precleaned supernatant was incubated with antibody needed for each specific experiment overnight. After incubation with 20 µl of protein G-Sepharose beads for 1 h, the suspension was centrifuged, and pellets were washed and collected as immunoprecipitation complexes. Western blotting was done as previously described (31, 49–51). Images were recorded using a luminescent image analyzer, and the intensities of the bands were quantitated by densitometry (NIH Image J software). Each protein was analyzed in samples from at least three independent experiments from each set of tumor cells, CICs and CAFs.

### Lipid-Raft Isolation

All procedures were done at 4° C. Cells were scraped into buffer containing 1% cold Triton X-100 buffer (20 mM Tris-HCl, pH 7.4, 150 mM NaCl, 1% Triton X-100, 1% deoxycholate, 0.1% sodium dodecyl sulfate [SDS], 1 mM EGTA, 1 mM sodium orthovanadate with a cocktail of protease inhibitors at final concentrations of 0.2 mM aminoethyl-benzene sulfonyl fluoride, 1 µg/ml aprotinin, 3 µM E-64, 10 µg/ml leupeptin, 2 µM pepstatin, and 50 µg/ml calpain inhibitor I) and lysed on ice for 30 minutes. After centrifugation at 800 x g to remove nuclei and cell debris, lysates were subjected to sucrose gradient fractionation as described previously (52). An equal volume of each fraction was boiled in SDS-Lammeli sample buffer and subjected to western blotting analyses as described previously (30, 31, 49, 50, 53, 54). On the other hand, the Triton-insoluble rafts and Triton-soluble fractions were diluted with an equal volume of extraction buffer (25 mM HEPES, pH 7.6, 0.3 M NaCl, 1.5 mM MgCl2, 0.2 mM EDTA, 1% Nonidet P-40, and 0.5 mM dithiothreitol) and subjected to immunoprecipitation as described previously (31, 49, 55, 56).

### Biotin Labelling of Cell Surface Receptors and Internalization Assays

For cell surface protein labelling, cells were treated in the presence or absence of FOLFOX or WNT3A conditioned media at 37°C for the times indicated and washed three times with ice-cold phosphate-buffered saline (PBS; pH 8.0) to remove any contaminating proteins. Biotinylation followed by immunoprecipitation and western blotting was done as described in our companion paper (48). For internalization assays, cell surface proteins were biotin-labelled as described in our companion paper (48). The amounts of receptor bound to beads were determined by SDS–PAGE and immunoblot analysis.

### Transient Transfection and Luciferase Reporter Assay

For the transient assays, 1.0 x 10^5^ cells were transfected using Lipofectamine LTX 2000 (Invitrogen) with 1 μg of each Luciferase construct and 100 ng of pRL-SV40 vector (Promega), according to the manufacturer’s instructions. Firefly and Renilla Luciferase activities were measured in cell lysates 48 hours after transfection using the DualGlo Luciferase Assay System (Promega) on a Veritas TM Microplate Luminometer (Perkin-Elmer) following the manufacturer’s protocol and as shown in our companion paper and as described previously (48, 55). All experiments were done in triplicate. Ratios of Renilla luciferase readings to firefly luciferase readings were taken for each experiment, and triplicates were averaged. The average values of the tested constructs were normalized to the activity of the empty pGL3-basic vector, which was arbitrarily set at value 1.

### β-Catenin/TCF Reporter Assays

All reporter gene assays were done in 96-well plates. PD-FR/CICs or CD44v6 overexpressing SW480-FR/SQ/Non-CICs (Non-CICs/CD44v6) (1.0 × 10^4^/well) were transfected with Super TOPFlash reporter (25 ng) and TK-Renilla (5 ng), and with the respective plasmid DNA as indicated using Lipofectamine™ 3000 transfection reagent according to the manufacturer’s protocol. Each transfection was adjusted to 150 ng DNA/transfection with pcDNA3.1 empty vector. Where indicated, cells were transfected at 50–70% confluency with shRNA constructs using Lipofectamine™ 3000 transfection Reagentin 6 cm petri dishes according to the manufacturer’s protocol 24 h before seeding the cells for the reporter assays. 50 ng/ml of WNT3A was added 24 h after DNA transfection. Cells were lysed 72 h after DNA transfection with 1 × Passive Lysis Buffer (Promega), and the luciferase activity was measured using the Luminescence counter (PerkinElmer). TOPFLASH experiments were normalized to co-transfected Renilla gene expression. In parallel to the reporter assay, transfected CICs and Non-CICs/CD44v6 cells were subjected to western blotting analysis to detect the proteins involved in CD44v6-*β*-catenin-MDR1 signaling.

### RNA Extraction and cDNA Synthesis

RNA extraction and cDNA synthesis were done following published work (57). Total RNA was isolated from cells using the RNeasy mini kit (Qiagen) according to the standard protocol provided by the manufacturer, with on-column DNA digestion. Five hundred ng of RNA was used for cDNA synthesis. One μl primer, 1 μl buffer (from 10X ezDNase™ Buffer), 0.5 μl RNase inhibitor, 1 μl dNTP (10 mM) and 0.5 μl Reverse Transcriptase (Thermo Fisher Scientific) and 6 μl Nuclease-free Water were mixed in a microtube (0.2 ml) to make 10 μL DNA digestion reaction mix for each RT-PCR reaction for cDNA synthesis. The synthesis was done at 50° C for 60 minutes in a thermal cycler (Bio Rad).

### Primer Design and Semiquantitative RT-PCR

Design of primers and semiquantitative RT-PCR were performed following published works (55, 57, 58). Primers were designed by online Primer Quest Tool (https://www.idtdna.com/PrimerQuest/Home/Index). The quality of designed primers was analyzed by Oligoaniline Tool software. The semi-quantitative PCR primer sequences used for CD44 exon specific PCR are given in **Tables 1**, **2**. The semiquantitative PCR primer sequences used for proteins and cytokines of CAFs are presented in **Table 3**. Semi-quantitative PCR was done using different amounts of cDNA of RNA samples. One μl of forward (F) and of reverse (R) primers were used. For each sample, PCR was repeated three times. The reaction contained 1 μl of each cDNA sample, 0.5 μl of each primer, 5 μl Taq DNA Polymerase 2× Master Mix Red (Amplicon Co.) and 3 μl dd water in a final volume of 10 μl. Before the main reactions, the PCR conditions, including thermal conditions, and the number of cycles and the cDNA concentrations, were optimized. During the main PCR cycles, temperature conditions included one initial denaturation cycle (3 min at 95° C) followed by 35 cycles with a denaturation step for 5 sec at 95°C and a combined annealing and extension step for 35 sec at 61°C. The PCR products were electrophoresed on agarose 2.5%, stained with ethidium bromide and photographed. The analysis of band intensities was done by ImageJ software.

**Table 1:** CD44 exon specific PCR examined using 5' primers complementary to individual variable exons and a primer to the 3' constitutive exon 7.

Genes	Accession number	Primers	
		Forward sequence (5'-3')	Reverse sequence (5'-3')
C_5_		AAGACATCTACCCCAGCAAC
C_7_			TTTGCTC CACCTTCTTGACTCC
h-CD44V2	NM_001001389.2	GAT GAG CAC TAG TGC TAC AG	TTTGCTCCACCTTCTTGACTCC
h-CD44V3	NM_001001390.2	ACG TCT TCA AAT ACC ATC TC	TTTGCTCCACCTTCTTGACTCC
h-CD44V4	NM_001001391.2	TCA ACC ACA CCA CGG GCT TT	TTTGCTCCACCTTCTTGACTCC
h-CD44V5	NM_00100139.2.2	GTA GAC AGA AAT GGC ACC AC	TTTGCTCCACCTTCTTGACTCC
h-CD44V6	NM_001202555.2	CAG GCA ACT CCT AGT AGT AC	TTTGCTCCACCTTCTTGACTCC
h-CD44V7	NM_001202556.2	CAG CCT CAG CTC ATA CCA G	TTTGCTCCACCTTCTTGACTCC
h-CD44V8	NM_001202557.2	TCC AGT CAT AGT ACA ACG CT	TTTGCTCCACCTTC TTGACTCC
h-CD44V9	XM_011520485.2	CAG AGC TTC TCT ACA TCA CA	TTTGCTCCACCTTCTTGACTCC
h-CD44V10	XM_005253238.3	GGT GGA AGA AGA GAC CCA AA	TTTGCTCCACCTTC TTGACTCC
h-C044C5V6		ATCCCTGC TACCATCCAGGCAAC	TTTGCTCCACC TTCTTGACTCC
h-CD44s	X155 150 (EMBL/Genebank)	AAGACATCTACCCCAGCAAC	TTTGCTCCACCTTCTTGACTCC
h-GAPDH	NM_002046.7	ACC ACA GTC CAT GCC ATC A	TCC ACC ACC CTG TTG CTG TA

**Table 2:** CD44 exon specific PCR examined using 3' primers complementary to v6 and vs exons and a primer to the 5' constitutive exon 5.

Genes	Accession number	Primers	
		Forward sequence (5'-3')	Reverse sequence (5'-3')
C_5_		CATCCCAGACGAAGACAGTC	
h-CD44V6	NM_001202555.2		CAG GCA ACT CCT AGT AGT AC
h-CD44V8	NM_001202557.2		GTIGTCATTGAAAGAGG TCCT
h-CD44s	X155150 (EMBL/Genebank)	CATCCCAGACGAAGACAG TC	TTIGCTCCACCTTCTTGACTCC
h-GAPDH	NM_002046.7	ACC ACA GTC CAT GCC ATC A	TCC ACC ACC CTG TIG CTG TA
C_7_			TTIGCTCCACCTTCTTGACTCC

**Table 3:** Semiquantitative RT-PCR primers for Cytokines, growth factors, PN and relates receptors.

Genes	Accession number	Primers	
		Forward sequence (5'-3')	Reverse sequence (5'-3')
IL-17A	NM_002190.3	AGATTACTACAACCGATCCACCT	GGGGACAGAGTTCATGTGGTA-
IL-17R	NM_014339.7	AGTTCCACCAGCGATCCAAC	GTCTGAGGCAGTCA TIGAGGC
I L-23A	NM_016584.3	CTCAGGGACAACAGTCAGTIC	ACAGGGCTATCAGGGAGCA
IL-23R	NM_ 144701.3	ACATGCTTCTATGTACTGCACTG	TGTGTCTATGTAGGTGAGCTICC
RORα	NM_134261.3	CTTGCCGTAGGGATGTCTCG	GAAGTTCCGTCAGC CCGTI
Periostin	NM_006475.3	CTCATAGTCGTATCAGGGGTCG	ACACAGTCGTITICTGTCCAC
lntegrin b1	NM_002211.4	CCTACTTCTGCACGATGTGATG	CCTTTGCTACGGTTGGTTACATI
PDGF α	NM_002607.6	GCAAGACCAGGACGGTCATTI	GGCACTTGACACTG CTCGT
TGFβ1	NM_000660.7	GGCCAGATCCTGTCCAAGC	GTGGGTTTCCACCATIAGCAC
TGFβ2	NM_001135599.4	CCATCCCGCCCACTTTCTAC	AGCTCAATCCGTIGTTCAGGC
G-CSF	X03438.1	GCTGCTTGAGCCAACTCCATA	GAACGCGGTACGACACCTC
WNT3A	NM_033131.4	AGCTACCCGATCTG GTGGTC	CAAACTCGATGTCCTCGCTAC
WNT5A	NM_003392.7	ATTCTTGGTGGTCGCTAGGTA	CGCCTTCTCCGATGTACTGC
SDF-1	AY874118.1	ATTCTCAACACTCCAAACTGTGC	ACTTTAGCTTCGGGTCAATGC
EGF	NM_001963.6	TGGATGTGCTIGATAAG CGG	ACCATGTCCTTTCCAGTGTGT
HGF	NM_000601.6	GCTATCGGGGTAAAGACCTACA	CGTAGCGTACCTCTGGATTGC
FGF	NM_000800.5	ACACCGACGGGCTTITATACG	CCCATTCTTCTIGAGGCCAAC
IL1β	NM_000576.3	AGCTACGAATCTCCGACCAC	CGTTATCCCATGTGTCGAAGAA
IL4	NM_000589.4	CCAACTGCTICCCCCTCTG	TCTGTIACG GTCAACTCGGTG
IL8	M28130.1	TTTTGCCAAGGAGTG CTAAAGA	AACCCTCTG CACCCAGTITIC
TNFα	NM_000594.4	CCTCTCTCTAATCAGCCCTCT	GAGGACCTGGGAGTAGATGAG
VEGFA	NM_001171623.2	AGGGCAGAATCATCACGAAGT	AGGGTCTCGATTGGATGGCA
IFNγ	NM_000619.3	TCGGTAACTGACTTGAATGTCCA	TCGCTTCCCTGTITIAGCTGC
PGE2	NM_004878.5	GTGACCGAGTICGG CAATAAG	CGGACAATGTAGTCAAAGGACG
IGF1	NM_001111283.3	GCTCTTCAGTICGTGTGTGGA	GCCTCCTIAGA TCACAG CTCC
IGF1R	NM_000875.5	TCGACATCCGCAACGACTATC	CCAGGGCGTAGTIGTAGAAGAG
CD44v6	NM_001202555.2	CTGCCGCTTTGCAGGTGTA	CATTGTGGG CAAGGTGCTATI
COX2	M90100.1	TAAGTGCGATIGTACCCGGAC	TTTGTAGCCATAGTCAGCA TIGT
FLT	NM_002019.4	GAAAACGCATAATCTG GGACAGT	GCGTGGTGTGCTTATTTGGA
MCP-1	NM_002982.4	CAGCCAGATGCAATCAATGCC	TGGAATCCTGAACCCACTICT
STAT3	NM_139276.3	CAGCAGCTTGACACACGGTA	AAACACCAAAGTGGCATGTGA
GAPDH	NM_002046.7	GGAGCGAGATCCCTCCAAAAT	GGCTGTTGTCATACTTCTCATGG

### Primer Design and Quantitative Real-Time RT–PCR

Design of primers and RT–qPCR were done following previously described protocols (55, 58). Total RNA was isolated from cells after various treatments and transfections as described in the figure legends for each specified experiment using the RNeasy mini kit (Qiagen) according to the standard protocol provided by the manufacturer, with on-column DNA digestion. RNA integrity and concentration were analyzed using Bioanalyzer, and 100 ng of RNA was retrotranscribed into cDNA using the First Strand cDNA synthesis kit from Roche Applied Science (Qiagen). SYBR Green technology (Bio-Rad) was used for all real-time PCR experiments. Amplification was done with the real-time PCR analyzer (Bio-Rad). The PCR mixture (25 µl) contained 12.5 µl of 2 SYBR Green PCR Master Mix (Bio-Rad), 5 µl of diluted RT product (1:20), and 0.5 µM sense and antisense primer sets. The real-time PCR assays were done in three individual experiments with duplicate samples using standard conditions in a CFX96 real-time PCR detection machine. After incubations at 95° C for 3 minutes, the amplification protocol consisted of 50 cycles of denaturing at 95° C for 10 sec, followed by annealing and extension at 60° C for 30 sec. The standard curve was made from a series dilution of template cDNA. Expression levels of tested genes were calculated after normalization with the housekeeping gene *GAPDH* or β*-*actin. The QPCR primers used in this study in analyses of various genes associated with fibroblast specific markers as well as for PN, IL17A and WNT3A, are presented in **Table 4**. The QPCR primers used in this study in analyses of various genes associated with CIC stemness factors are presented in **Table 5**.

**Table 4:** Real-time PCR (QPCR) primers for various genes used in this study.

Genes	Accession number	Primers	
		Forward sequence (5'-3')	Reverse sequence (5'-3')
IL17A	NM_002190.3	AAGACCTCATTGGTGTCACTGCTAC	ATCTCTCAGGGTCCTCATTGCG
Periostin	NM_006475.3	TGTTGCCCTGGTTATATGAG	ACTCGGTGCAAAGTAAGTGA
WNT3A	NM_033131.4	GGATACTTCTTACTCCTCTGCAG	AATGGCGTGGACAAAGGCCGACT
PDGFR α	NM_006206.6	GGTGGTCACAGGTG	CTTAAGGCTCTCAGGA
EpCAM	NM_002354.3	GCCAGTGTACTTCAGTTGGTGC	CCCTTCAGGTTTTGCTCTTCTCC
FAP	NM_004460.5	TGTGCATTGTCTTACGCCCT	CCGATCAGGTGATAAGCCGT
FSP1	NM_002961.3	TTGGGGAAAAGGACAGATGAAG	TGAAGGAGCCAGGGTGGAAAAA
α-SMA	NM_001100.4	CTATGCCTCTGGACGCACAACT	CAGATCCAGACGCATGATGGCA
GAPDH	NM_002046.7	GAAGGTGAAGGTCG	CTTCCCGTTCTCAG
β-actin	NM_001904.4	AGAAAATCTGGCACCACACC	AGAGGCGTACAGGGATAGCA

**Table 5:** Real-time PCR (QPCR) primers for various genes associated with CICs stemness functions.

Genes	Primers	
	Forward sequence (5’–3’)	Reverse sequence (5’–3’)
SOX-2	GGACTGAGAGAAAGAAGAGGAGAG	CGCCGCCGATGATTGTTATTA
OCT4	GGAGGAAGCTGACAACAATGA	CTCTCACTCGGTTCTCGATACT
c-MYC	AAGCTGAGGCACACAAAGA	GCTTGGACAGGTTAGGAGTAAA
TWIST1	AGACTCTGGAGCTGGATAACT	GCCTGTCTCGCTTTCTCTTT
ALDH1	CTTGGAATTTCCCGTTGGTTATG	GAGAGCAGTGAGAGGAGTTTG
MDR1	TGCTGGTTGCTGCTTACA	GCCTATCTCCTGTCGCATTATAG
CD44v6	GACAGAATCCCTGCTACCAATAG	TCCTTCGTGTGTGGGTAATG
GAPDH	GAAGGTGAAGGTCG	CTTCCCGTTCTCAG
β-actin	AGAAAATCTGGCACCACACC	AGAGGCGTACAGGGATAGCA

Validation of pSico-CD44v6shRNA in Cells In order to use shRNA for target genes in in vivo experiments, pSicoR-CD44v6 shRNA, pSicoR-WNT3A shRNA, and pSicoR- b-catenin shRNA were prepared as described in our previous study (59) in the RNA silencing section. The abilities of pSico and pSicoR vectors to conditionally silence endogenous CD44v6 genes were demonstrated by their ability to inhibit expression of the human CD 44 v6 expressionin SW 480- FR cells (**Figures 9A–C**, and **Figures 9D–F**). PCR was done to amplify the recombined and unrecombined genomic plasmid DNAs from SW480-FR cells.

### RNA Silencing and Confirmation of the Specificity of shRNA

For determining shRNA sequences, coding nucleotide sequences were used of the genes obtained from the National Institutes of Health database, website (www.ncbi.nlm.nih.gov). The hairpin shRNAs to target transcript sequences were designed using the Broad Institute GPP Web Portal (http://portals.broadinstitute.org/gpp/public/). The sequences for cloning in pSico/pSicoR vectors were designed following instructions described in the Jackson Lab website (http://web.mit.edu/jacks-lab/protocols). The resulting pSicoR-CD44v6 shRNA1 (CD44v6 sh1), pSicoR-CD44v6 shRNA2 (CD44v6 sh2), pSicoR-WNT3A shRNA1 (WNT3A sh1), pSicoR-WNT3A shRNA2 (WNT3A sh2) transfectants constitutively silence respectively CD44v6, WNT3A and β-catenin RNAs in the cells. The pSicoR-Non targeted shRNA (NT sh) was used as a control to the above shRNA transfectants for shRNA sequences used in this study. The specificities of the prepared shRNAs were confirmed (31, 49) as described in our companion paper (48). The primers for various shRNAs used in this study are given in **Table 6**.

**Table 6:** shRNA sequence in pSico and pSicoR vectors (https://web.mit.edu/jacks-lab/protocols/).

Genes	Primers	
	Sense sequence (5’–3’)	Antisense sequence (5’–3’)
CD44v6 shRNA1	TCCTCCCAGTATGACACATATTTTCAAGAGAAATATGTGTCATACTGGGAGGTTTTTTC	TCGAGAAAAAACCTCCCAGTATGACACATATTTCTCTTGAAAATATGTGTCATACTGGGAGGA
CD44 shRNA2	T GGACCAATTACCATAACTATTTCAAGAGA AATAGTTATGGTAATTGGTCCTTTTTTC	TCGAGAA AAAAGGACCAATTACCATAACTATTTCTCTTGAAAATAGTTATGGTAATTGGTCCA
WNT3A shRNA 1	TGTAGCGAGGACATCGAGTTTGTTCAAGAGACAAACTCGATGTCCTCGCTACTTTTTTC	TCGAGAAAAAAGTAGCGAGGACATCGAGTTTGTCTCTTGAACAAACTCGATGTCCTCGCTACA
WNT3A shRNA 2	TGAACTACGTGGAGATCATGCTTCAAGAGAGCATGATCTCCACGTAGTTCCTTTTTTC	TCGAGAAAAAAGGAACTACGTGGAGATCATGCCTCTTGAACATGATCTCCACGTAGTTCCA
βcatenin shRNA 1	TATCTGTCTGCTCTAGTAATAATTCAAGAGATTATTACTAGAGCAGACAGATTTTTTTC	TCGAGAAAAAAATCTGTCTGCTCTAGTAATAATCTCTTGAATTATTACTAGAGCAGACAGATA
βcatenin shRNA 2	TTCTAACCTCACTTGCAATAATTTCAAGAGAATTATTGCAAGTGAGGTTAGATTTTTTC	TCGAGAAAAAATCTAACCTCACTTGCAATAATTCTCTTGAAATTATTGCAAGTGAGGTTAGAA
Caveolin-1 shRNA1	TACCTTCACTGTGACGAAATATTCAAGAGA TATTTCGTCACAGTGAAGGTGTTTTTTC	TCGAGAAAAAACACCTTCACTGTGACGAAATATCTCTTGAATATTTCGTCACAGTGAAGGTGA
Caveolin-1 shRNA2	T ACCTTCACTGTGACGAAATATTCAAGAGA TATTTCGTCACAGTGAAGGTG TTTTTTC	TCGAGAAAAAAATCAACTTGCAGAAAGAAATATCTCTTGAATATTTCTTTCTGCAAGTTGATA
Clathrin shRNA1	TTGACTATGGAGTCTGACAAATTTCAAGAGA ATTTGTCAGACTCCATAGTCA TTTTTTC	TCGAGAAAAAATGACTATGGAGTCTGACAAATTCTCTTGAAATTTGTCAGACTCCATAGTCAA
Clathrin shRNA2	TACTATGGAGTCTGACAAATTTTCAAGAGA AATTTGTCAGACTCCATAGTC TTTTTTC	TCGAGAAAAAAGACTATGGAGTCTGACAAATTTCTCTTGAAAATTTGTCAGACTCCATAGTCA
Firefly luciferase shRNA1	TGCCCTGGTTCCTGGAACAATTTTCAAGAGA AATTGTTCCAGGAACCAGGGCTTTTTTC	TCGAGAAAAAAGCCCTGGTTCCTGGAACAATTTCTCTTGAAAATTGTTCCAGGAACCAGGGCA
Firefly luciferase shRNA2	TTGAGTATTTCTGTCTGATTTTTCAAGAGA AATCAGACAGAAATACTCAC TTTTTTC	TCGAGAAAAAAGTGAGTATTTCTGTCTGATTTTCTCTTGAAAATCAGACAGAAATACTCACA

### Chromatin Immunoprecipitation (ChIP) Assay

The chromatin immunoprecipitation (ChIP) assay was done using the ChIP assay kit (Upstate Biotechnology) following the manufacturer’s directions (44). Details of the ChIP assay and the primers used for ChIP PCR studies are presented in **Table 7**, and were described in our companion paper (48).

**Table 7:** ChIP PCR primers for MDR1 and CD44v6 promoters.

Genes	Primers	
	Forward sequence (5’–3’)	Reverse sequence (5’–3’)
MDR1 (A) [-644- (-447)]	TAGGTCTTTCCACTAAAGTC	AGAGGACTTCACACTATCCA
MDR1 (B) [-1218- (-980)]	TTTCTTTCATTCCATTTATC	AAGTCTTCATATCCATATAA
MDR1 (C) [-1301- (-1056)]	AATGTAAGAATTTAAAATGC	CTTTGAAAAGGCTAGGAGAA
CD44v6 (A) [-1618- (-1370)]	AGAAGTCCTGGCATGGTTCC	TCTTCAGGGGAAGCCTTTTGA
CD44v6 (B) [-1997- (-1793)]	GGATGACTTACTTGTCCCTGT	ACTCACAAGCAGGCCATTACCA

### *In Vivo* Tumorigenic Potential of the CAFs to Affect the Tumorigenic Capacity of CICs

CICs and CAFs were isolated from subcutaneous SW480-FR/SQ xenografts in SCID mice (using an approved IACUC protocol). Tumorigenic potentials of CICs alone or in combination with CAFs (DMSO or FOLFOX treated) were determined by subcutaneous implantation in the flanks of six-week-old SCID female mice from the Jackson Laboratory. The CAFs were treated with DMSO or FOLFOX (50 μM 5-FU + 10 μM OXA + 1 μM leucovorin) for 3 days. Tumorigenic potentials of CICs alone (4 x 10 ^3^), or CICs (4 x 10^3^) + CAFs (4 x 10^4^) pre-treated with DMSO, or CICs (4 x 10^3^) + CAFs (4 x 10^4^) pre-treated with FOLFOX were determined by SQ implantation as described above. Twenty-five mice per cell types were used. The appearances of tumors were monitored, and five mice were sacrificed every 2 weeks. Tumors were removed and weighed to evaluate the tumor development (**Figure 8**).

### *In Vivo* Evaluation of the CIC Tumor Growth Dependence on PN, WNT3A, IL17A and CD44v6

CICs (2 x 10^4^) and CAFs (6 × 10^4^) together were implanted subcutaneously (SQ) into immunocompromised mice (using an approved IACUC protocol). When tumors reached ~0.3 cm^3^ in volume, treatment was initiated. Four arms were included: 1) isotype control (2 mg/kg antibody, 3 times/wk for 2 wks followed by 1 time/wk for 2 wks); 2) FOLFOX chemotherapy alone (1x FOLFOX, 3 times/wk for 2 wks, followed by 1 time/wk for 2 wks); 3) PN, or IL17A, or WNT3A, or CD44v6 blocking antibody therapy alone (2 mg/kg antibody, 3 times/wk for 2 wks, followed by 1 time/wk for 2 wks); and 4) a combination of the blocking antibodies and FOLFOX chemotherapy (5 mice per each time point, 0 wk, 2 wks, 4 wks and 6 wks). Mice were weighed, and the tumor volumes were measured by caliper every other day for 6 weeks. Every two weeks mice were sacrificed, and tumors were collected.

### Conditional Knockdown of CD44v6

To test whether CD44v6 is a good therapeutic target, an *in vivo* approach to deliver shRNA specifically targeting CD44v6 was developed. Two vectors (floxed pSico-CD44v6shRNA + pFabpl-Cre) were engineered and encapsulated into transferrin (Tf)-coated nanoparticles (59, 60). The shRNA vectors are inherently inactive due to the presence of a Lox-Stop-Lox cassette prior to the start codon. When injected intraperitoneally (i.p.), there is ubiquitous cellular uptake of these vectors. However, only cells expressing FABPL (i.e. intestinal epithelium specific) will express the Cre protein and activate the shRNAs. First, we prepared transferrin (Tf)-PEG-PEI nanoparticles and encapsulated both plasmids (floxed pSico-CD44v6shRNA + pFabpl-Cre) into them following our published procedure (59). Our pSico-CD44v6shRNA in SW480-FR cells was validated in **Figure 9**.

### Preparation of Transferrin (Tf)-Coated PEG-PEI (Nanoparticle)

The transferrin (Tf)-PEG-PEI/Nanoparticle (Nano) was prepared as validated in our previous studies (31, 49, 59). Briefly, transferrin was linked with N*-*hydroxy succinimide/PEG/maleimide and then allowed to react with a mercaptopropionate-modified branched PEI to form Tf-PEG-PEI (59). The pSico-CD44v6 shRNA, or pFabpl-Cre plasmid were jointly encapsulated in the purified Tf-PEG-PEI conjugate (size: ~80 ± 31 nm).

### Validation of pSico-CD44v6shRNA in Cells

In order to use shRNA for target genes in *in vivo* experiments, pSicoR-CD44v6 shRNA, pSicoR-WNT3A shRNA, and pSicoR-β-catenin shRNA were prepared as described in our previous study (59) in the RNA silencing section. The abilities of pSico and pSicoR vectors to conditionally silence endogenous CD44v6 genes were demonstrated by their ability to inhibit expression of the human CD44v6 expressionin SW480-FR cells (**Figures 9A–C**, and **Figures 9D–F**). PCR was done to amplify the recombined and unrecombined genomic plasmid DNAs from SW480-FR cells.

### *In Vivo* Distribution of shRNA in Tissues

Distributions of shRNA against the firefly luciferase gene (pSico-Firefly luciferase shRNA/Nano) plus pFabpl-Cre/Nano were determined in various organs of C57Bl/6 mice (n = 4) that were previously injected (i.p.) with reporter plasmids expressing firefly luciferase and renilla luciferase. Twelve hours after their injection, pSico-Firefly luciferase shRNA/Nano plus pFabpl-Cre/Nano were injected (i.p.) Thirty-six hours later, cells from SQ tumors, intestine, colon, kidney and liver tissues were lysed, and the ratios between firefly and renilla luciferase activities were calculated. Distributions of the tissue specific delivery of luciferase-shRNAs were determined by measuring activities of luciferase-shRNA in various tissues as indicated above. Three independent experiments were performed. Western blot analyses of extracts from the various treated tumors collected at the end of the experiment were analyzed for CD44v6 and MDR1 expressions (*β*-tubulin was used as an internal standard).

### *In Vivo* Targeting of CD44V6 by Genetic Modification to Inhibit CICs Tumor Growth in Mice

Study design: 2 x 10^4^ CICs and 6 × 10^4^ CAFs were isolated from SW480-FR SQ tumors, and PD-FR tumor specimens were implanted together into 8-week-old female SCID mice and NSG mice. When tumors reached ~0.3 cm^3^ in volume, treatment was initiated. Ten arms were included: Group 1: [(100 µg) of pSico NT shRNA-Nano (3 x per wk for 4 wks)]; Group 2: [(100 µg) of pFabpl Cre-Nano (3 x per wk for 4 wks)]**;** Group 3: 1 x FOLFOX [(3 x per wk for 4 wks)] with pSico NT shRNA-Nano (3 x per wk for 4 wks)]**;** Group 4: 1 x FOLFOX [(3 x per wk for 4 wks] with pFabpl Cre-Nano (3 x per wk for 4 wks); Groups 5, 7, and 9: (pSico v6 shRNA plus Fabpl Cre)-Nano [(each plasmid 20 µg, Group 5); (each plasmid 50 µg, Group 7); (each plasmid 100 µg, Group 9) (3 x per wk. for 4 wks)]; Groups 6, 8 and 10: ([pSico v6 shRNA plus Fabpl Cre]-Nano + 1 x FOLFOX [3 x per wk for 4 wks]); [(each plasmid 20 µg plus FOLFOX, Group 6); (each plasmid 50 µg plus FOLFOX, Group 8); (each plasmid 100 µg plus FOLFOX, Group 10) (3 x per wk. for 4 wks)]. Seven SCID mice per group were used. Mice were weighed every other day, and the tumor volumes were measured by caliper every day. Mice were sacrificed every week for 4 weeks, and tumor weights were taken.

### Statistics

A two-tailed Student’s t-Test was used to compare mean value between sensitive and resistant cells using the following parameters: mean ΔΔCT values for QPCR; mean colony number for soft agar growth assays; mean densitometry values for QPCR and WB; mean percentage of cell viability assay (CellTiter-Glo) and FACS analysis; mean luminescence for ATP activity in cell growth, Caspase Glow assays in Apoptosis measurements; and mean tumor weight in xenograft studies. Chi-squared analysis was used to compare incidences between sensitive and resistant cells for the following assays: number of positive wells containing tumor spheres in the sphere formation assay, and numbers of mice developing tumors in xenograft studies. For experiments involving three or more groups, statistical significance was calculated with GraphPad Prism Software (version 8) using a 1-way or 2-way ANOVA with a Bonferroni’s posttest, Student’s *t* test, or log-rank (Mantel-Cox) test where appropriate (Graph-Pad Software Inc.). Data are represented as the mean ± SD.

### Ethics Statement

The animal study was approved by the Institutional Animal Care and Use Committee (IACUC) at the Medical University of South Carolina (MUSC). Procedures for animal studies were conducted in accordance with the National Institutes of Health Guide for the Care and Use of Animals IACUC-2017-00250 (approval date: 2019/03/14-2021/03/29).

### Data Availability Statement

All the data are included within the manuscript. The names of the repository/repositories and accession number(s) can be found in the article supporting information. Coding nucleotide sequences of the genes were obtained from the NCBI, National Institutes of Health, website (www.ncbi.nlm.nih.gov). Hairpin shRNAs were designed to target a transcript sequence using the Broad Institute GPP Web Portal (http://portals.broadinstitute.org/gpp/public/). Primers were designed by online Primer Quest Tool (https://www.idtdna.com/PrimerQuest/Home/Inde.

## Results

### FOLFOX-stimulated Enrichment of CAFs Promotes Expression of CD44v6, Which Defines Highly Tumorigenic Potential of CICs

**Figure 1:**
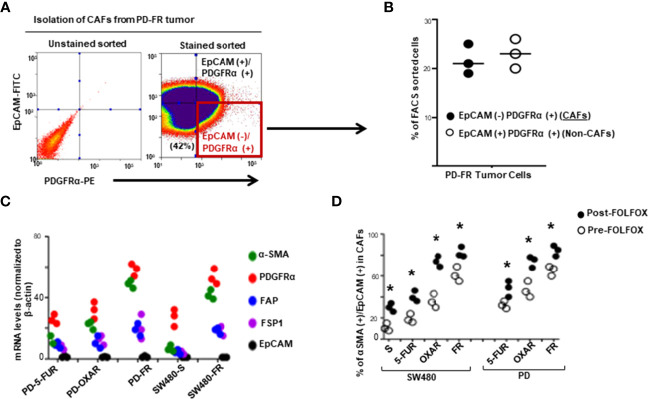
Cancer associated fibroblasts (CAFs) account for tumor resistance to FOLFOX. **(A)**, Single cell suspensions isolated from a dissociated colorectal tumor (PD-FR) from a FOLFOX resistant (FR) patient were sorted by FACS using PDGFR-α-PE and EpCAM-FITC. CAFs were analyzed by FACS using: (i) the epithelial marker EpCAM-FITC; and (ii) the functional fibroblast cell immunophenotype by PDGFR-α-PE. **(B)**, Percentages of EpCAM [-]/PDGFR-α [+] (CAFs) and of EpCAM [+] /PDGFR-α [+]) (Non-CAFs) in total unsorted cell populations were quantified. **(C)**, The integrities of CAFs (EpCAM [-]/PDGFR-α [+]) from the dissociated patient colorectal specimens (PD-5FUR, PD-OXAR, PD-FR), and from the sensitive and FOLFOX resistant SW480/subcutaneous (SQ) tumor tissues were confirmed by QPCR analyses for CAF-associated markers FSP1, FAP and PDGFR-α, and for epithelial cell marker EpCAM (negative control), and for ☐ SMA. **(D)**, CAFs from the PD- 5FUR, PD-OXAR, PD-FR, SW480-S and SW489-FR (SQ) tumors were cultured in DMEM with 10% BSA and were further sorted by FACS using α SMA-FITC and PDGFR-α-PE antibodies. The percentages of the sorted α SMA (+)/PDGFR-α [+] cells in total EpCAM [-]/PDGFR-α [+] cells were quantified. Data are presented as Mean ± SD from six independent replicates in three independent experiments. **(D)**, *, P < 0.05 was considered significant, percent CAFs with post-FOLFOX treatment compared with pre-FOLFOX-treated cells after normalization to CAFs of serum starved untreated SW480 controls. Student’s t-test was used to assess the significance.

The majority of studies indicate that cancer stem cells (CSCs) in solid tumors divide symmetrically, do not display multipotency, and are unable to generate an entire array of lineages (61). Subsequently, the CSCs are termed as cancer initiating cells (CICs) to define this subset of cells with self-renewal and tumorigenic potential (62, 63). Furthermore, the hierarchical model suggests that the CIC is the cell-of-origin of tumor cells. During cancer treatment, chemotherapy inflicts strong selective pressures on cancer cells to gain characteristics that promote the recruitment of pro-tumorigenic tumor microenvironment cells. Chemotherapeutics modulate the composition, or function of the tumor microenvironment cells thereby further altering the selective pressures to which cancer cells are exposed. This promotes resistance to apoptosis through the ECM adhesion proteins, integrins and CD44/CD44v6, which integrates cellular microenvironmental cues with stromal-secreted growth factors and cytokines including WNT, IL-6, IL17A, SDF-1, HGF, FGF, and TGF-ß leading to the activation of several tumor survival pathways (48). We therefore generated FOLFOX resistant (FR) CRC cells from their sensitive (S) pairs (see details in preparing these FR cells described in materials and methods and in our companion paper (48). Next, the responses of CAFS from biopsies of a group of colorectal patients resistant to 5-FU (PD-5FUR), Oxaliplatin (PD-OXAR), FOLFOX (PD-FR) and their sensitive pairs of CRC cells derived from SQ tumors were analyzed. Viable CAFs were isolated from freshly resected PD-colon tumor tissues and from SQ/FR and SQ/S tumor samples by FACS using platelet-derived growth factor receptor-α (PDGFR-α) and EpCAM antibodies (**Figure 1A**). Data in **Figure 1B** show that unsorted PD-FR cells contain ~20% of EpCAM (–)/PDGFRα(+) (hereafter referred to as CAFs) and slightly more than 20% EpCAM(+)PDGFR*α*(+) cells (henceforth referred to as Non-CAFs). The PD/SQ-derived and FR/SQ tumor-derived CAFs expressed high levels of fibroblast mRNA markers (via QPCR analyses) *α*SMA, PDGFR*α* and FAP, but very little or no epithelial cell marker EPCAM (**Figure 1C**). The data in **Figure 1C** also demonstrated that fibroblast markers, including *α*SMA and PDGFR*α*, increased in FOLFOX resistant (FR) cells. Moreover, the basal level of *α*SMA in all indicated CAFs isolated from chemo-resistant colon tumors significantly increased compared to CAFs isolated from the corresponding sensitive (S) SQ tumor cells (**Figure 1C**). **Figure 1D** shows quantitative measures of the fibroblast component *α*-SMA versus the epithelial component EpCAM with or without FOLFOX treatment (measured by FACS sorting). The results show that CAFs derived from SQ/FR tumor cells enrich the proportion of fibroblasts to the epithelial component (*α*SMA/EpCAM), compared to SQ/5-FUR, SQ/OXAR and SQ/S tumor cells. The *α*SMA versus EpCAM ratio is further increased with FOLFOX treated CAFS in all the tumor types (**Figure 1D**), confirming that CAFs are enriched in post-FOLFOX therapy on tumors.

### CD44v6 Expression Establishes a Highly Tumorigenic Colorectal Population

**Figure 2:**
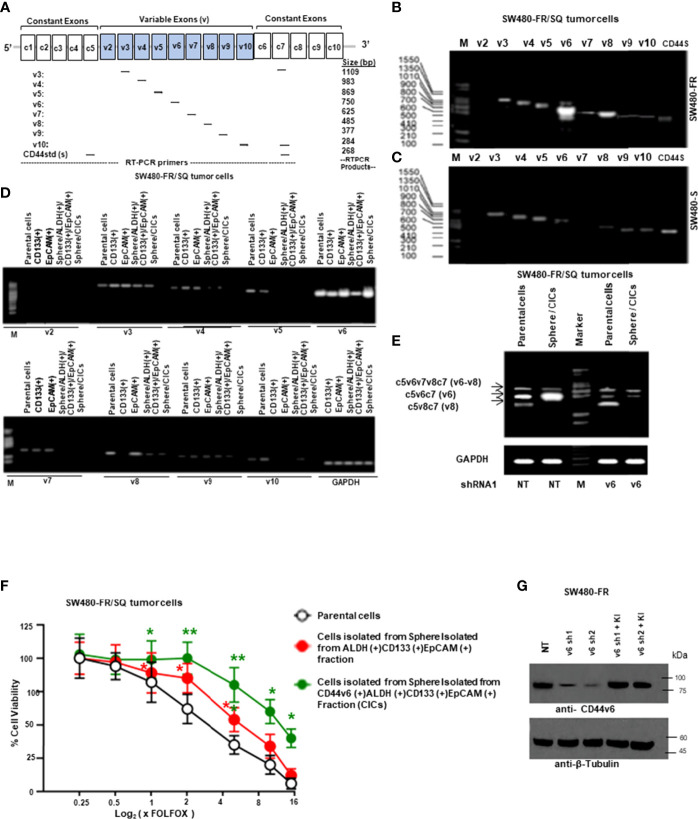
CD44v6 appeared considerably higher in FACS sorted CD133+, ALDH+ and EpCAM+ cells, and in sphere-propagated ALDH+/CD133+/EpCAM+ cells relative to bulk primary cells. **(A)**, Constant and variable exons are shown for the PCR primers used to amplify CD44 variable (v) and standard (s) isoforms in the human CD44 gene. Primer positions are shown. The expected sizes of the PCR products are indicated. The primers for both the CD44v6 and standard isoforms (CD44s) predominantly generate one PCR product (c5v6c7 [v6] for CD44v6, and C5C7 for CD44s), whereas the primers for the v8 variants amplify two splice variants C5v6v7v8C7 (v6-v8) and C5v8C7 (v8). These PCR products are depicted in panels **(B, E)** experiments. **(B)**, SW480-FR cells show a differential expression profile of CD44 isoforms when compared with that of the SW480-S cells **(C)**. **(D)**, RT-PCR analyses are shown of CD44 variants (v2-v10) in parental cells, freshly purified CD133+ cells, EpCAM+ cells, sphere-propagated FACS sorted ALDH1+CD133+EpCAM+, and CD44v6+ALDH1+CD133+EpCAM+ (sphere/CICs) from cells SW480-FR/SQ tumor cells. Normalization was done by using GAPDH as a housekeeping control gene. **(E)** RT-PCRs are shown for CD44v6, CD44v6-v8, and CD44v8 on Non-targeted control shRNA (NTshRNA) cells and on CD44v6shRNA1 overexpressed in parental cells and in sphere/CICs. GAPDH was used as a housekeeping control gene. **(F)**, FOLFOX sensitivity was measured in parental cells, in freshly isolated sphere/ALDH1+CD133+EpCAM+ cells, and in sphere/CICs isolated from SW480-FR/SQ tumors grown in serum free medium that were treated with various concentrations of FOLFOX. An ATP based assay (CellTiter-Glo) measured cell viability compared with control cells (without FOLFOX treatment) as 100%. Error bars represent calculated SDs (n = 3). **(G)** Validations of two shRNAs for CD44v6 used in panel **(E)** were done by the indicated shRNA mediated knockdown and the corresponding knock-in (KI) gene transfections as described in Methods. Target proteins were analyzed by WB analysis (β-tubulin, internal control). Data are presented as Mean ± SD from n = 3–5 independent replicates in three independent experiments. All semi quantitative RT-PCR and western blot data are representative of three experiments. *, P < 0.05, **, P < 0.01 were considered significant, percent cell viability in ALDH (+)/CD133 (+)/EpCAM (+), and from CD44v6 (+)/ALDH (+)/CD133 (+)/EpCAM (+) fractions compared with control parental cell. Student’s t-test was used to assess the significance.

It has been previously demonstrated that colorectal CICs are enriched in tumor spheres or in freshly fractionated ALDH(+), or CD133(+), or EpCAM(+) cells (48, 55, 64). Moreover, since ALDH, CD133 and EpCAM are well known colorectal CIC markers (55, 64–67), we determined whether a sphere-propagated CD44v6(+)ALDH(+)CD133(+)EpCAM(+) (CICs) subpopulation of cells demonstrate distinct drug resistance properties. Since our focus in this study is on CD44v6 function in mediating communication between CAFS-derived factors and CICs isolated from resistant and sensitive colorectal cells, first we compared the expressions of different CD44 variant isoforms in our sensitive and resistant SW480 cells and designed a series of forward primers, which base pair with v3, v4, v5, v6, v7, v8, v9 and v10 exons independently (**Figure 2A**). The reverse primer formed a base pair with a constant exon (c7). As shown in **Figures 2B, C**, CD44 exon specific RT-PCRs were examined using 5’ primers complementary to individual variable exons and a primer to the 3’ constitutive exon 7 (c7) (primers are shown in **Table 1**). The results demonstrated that although SW480-S and SW480-FR cells expressed similar CD44 isoforms, the CD44v6 isoforms in resistant cells are significantly higher compared to sensitive cells. In addition, as shown in **Figure 2D**, although several of the variable CD44 exons were uniformly translated across SW480-FR/SQ tumor subsets, mRNA levels of CD44v6 appeared considerably higher in bulk parental primary tumors cells, freshly purified CD133(+) cells, EpCAM(+) cells, Sphere-propagated FACS sorted ALDH1(+)CD133(+)EpCAM(+) cells (Sphere/ALDH1(+)/CD133+/EpCAM+) cells, and in Sphere-propagated FACS sorted CD44v6(+)ALDH1(+)CD133(+)EpCAM(+) cells (Sphere/CICs) from SW480-FR/SQ tumor cells (**Figure 2D**). Analysis of CD44v6-containing isoforms in these cells further suggested that CD44v6 is translated in the same subset of SW480-FR cells (**Figure 2D**). Intriguingly, CD44v6 is significantly expressed more in sphere-propagated cells than in their corresponding parental primary tumors cells (**Figure 2E**), and knocking down CD44v6 with specific shRNA downregulates all CD44v6-containing isoforms (**Figure 2E**, validation of CD44v6 shRNA is shown in **Figure 2G**). Since CD44v6, ALDH, CD133 and EpCAM are well known colorectal CIC markers (55, 64–67), analysis of FOLFOX sensitivity in Sphere/CICs, in Sphere-propagated CD44v6(+)/ALDH1(+)/CD133(+)/EpCAM(+) (Sphere/CICs) cells, and in Sphere-propagated ALDH1(+)/CD133(+)/EpCAM(+) cells were compared to parental SW480-FR cells (**Figure 2F**). Results in **Figure 2F** further suggested that Sphere/CICs are more resistant than in their corresponding Sphere/ALDH1(+)/CD133(+)/EpCAM(+) cells and in parental primary tumor cells. Taking into consideration; 1) the strong elevation of the expression of CD44v6 isoform in FR cells compared to sensitive cells (**Figures 2B, C**); and 2) that sphere/CICs located in tumor spheres are more resistant compared to Sphere/ALDH1(+)/CD133(+)/EpCAM(+) cells and primary parental tumor cells (**Figure 2F**), we concluded that CD44v6 splicing likely defines a colorectal CIC population with increased drug resistance properties.

**Figure 3:**
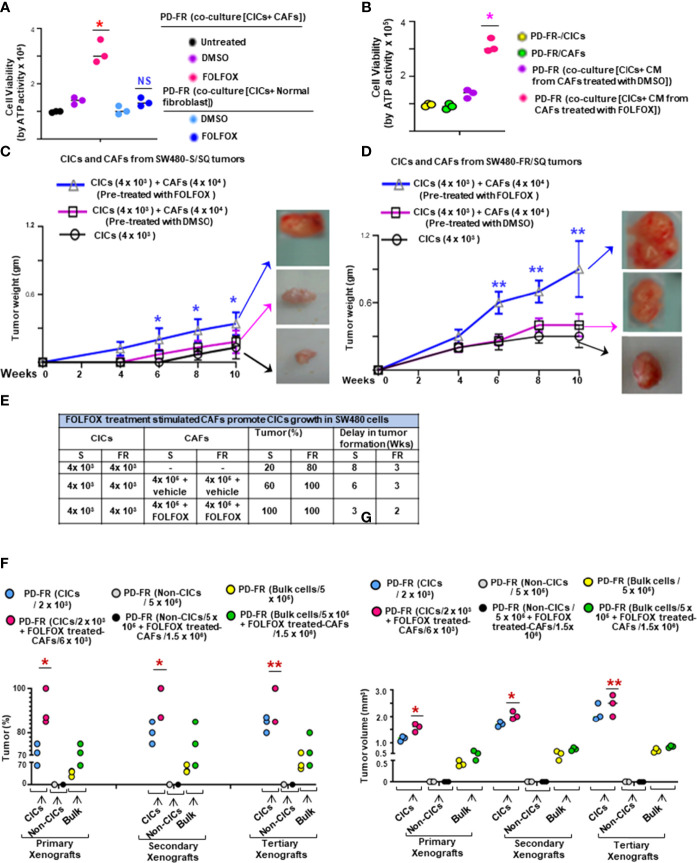
FOLFOX-stimulated CAFs promote CIC growth. (A, B), The effect of CAFs on CICs was measured by co-culture of PD-FR/CICs with PD-FR/CAFs (A) and conditioned media (CM) treatment **(B)**. **(A)**, 1.5 x 105 PD-FR CICs were cultured with 3 x 105 PD-FR CAFs or with 3 x 105 normal fibroblasts pre-treated with vehicle (DMSO) or with FOLFOX-therapy for 12 days. Cellular viability was measured by the ATP Glo assay. **(B)**, 1.5 x 105 PD-FR/CICs, and 3 x 105 PD-FR CAFs were treated with vehicle (DMSO) or with FOLFOX for 72 hours. Conditioned media (CM) from CAFs were collected and used to culture CICs for 12 days. Cellular viability was measured by the ATP Glo assay. **(C, D)**, The abilities of the CAFs (4 x 104 ) to affect the tumor growth of CICs (4 x 103 ) from three different batches of SW480-S. **(C)** and of SW480-FR **(D)** were tested for tumorigenic potential. SW480-FR/SQ CAFs that were pre-treated with vehicle (DMSO) or with FOLFOX for 72 hours were subcutaneously co-implanted in immunocompromised mice. Tumors were harvested every week to evaluate their latency, and weights were measured to evaluate their development. **(E)**, The contributions of SW480-S/CAFs and SW480-FR/CAFs to post-FOLFOX treated tumor growth (tumor %) in their CICs were assessed by measuring tumor 10 weeks after implantation of the CICs and CAFs into immunocompromised mice. **(F)**, Numbers of SQ tumors formed by injections of the indicated numbers of CICs, Non-CICs, and unfractionated bulk tumor cells with and without addition of FOLFOX treated CAFs from the PD-FR cells are shown. FACS sorted CICs (2 x 103), Non-CICs (5 x 105), and the unfractionated bulk tumor cells (5 x 105), with and without FOLFOX treated CAFs (6 x 103 or 1.5 x 106 cells) from PD-FR tumor cells were resuspended in Matrigel and implanted in immunocompromised mice. The same cells from the first generation of SQ tumors were further implanted into immunocompromised mice to generate second generation of xenograft tumors. The experiments were repeated to generate tumors into a third generation of xenograft tumors. Only CICs and the unfractionated bulk tumor cells were capable of inducing first generation xenograft tumors formations. Isolation of second and third generation xenograft tumors displayed similar results (n = 5 analyzed patients derived cells, 8 mice group and experiments were performed in triplicates), Tumor percent (percentage of tumor bearing mice) **(F)** and tumor volumes **(G)** from experiment **(F)** were evaluated. Data are presented as Mean ± SD from n = 5 analyzed patients derived cells, 8 mice/group and experiments independent replicates in three independent experiments. **(A, B)**, *, P < 0.05 was considered significant, percent cell viability with FOLFOX treatment compared with DMSO treated coculture after normalization to that of untreated coculture. Student’s t-test was used to assess the significance. **(C, D)**, Tumor growth stimulation data with pre-treatment with FOLFOX compared with DMSO-treated CICs + CAFs after normalization to tumor growth of CICs represent means ± SD.; n = 5-8; *, P < 0.05. **, P < 0.01 were considered significant; **(F, G)**, Tumor (%) and tumor volume of primary, or secondary, and tertiary xenografts from CICs-tumor compared to Bulk-tumor populations. *, P < 0.05, **, P < 0.01 were considered significant; ANOVA, followed by Bonferroni’s post-hoc test was used to assess the significance in experiments **(C, G)**

Given that FOLFOX significantly increased the percentage of CAFs (*α*SMA[+]/EpCAM[+]) after drug (FOLFOX) treatment (as seen in **Figure 1D**), next, we determined whether FOLFOX treatment induces microenvironmental signals to instruct the tumorigenic potential of CICs. Thus, the effects of CAFs on viability of CICs were determined in co-culture experiments in which CICs and CAFs were either in close contact or in proximity, but not in direct contact, and by co-culture with conditioned media (CM) from the CAFs. Co-culture with FOLFOX-treated PD-FR/CAFs, or with their CM increased the PD-FR/CIC viability compared with DMSO (vehicle)-treated control (**Figures 3A, B**). Thus, CM from FOLFOX-treated PD-FR/CAFs contains secreted factors that promote PD-FR/CIC growth independent of contact. CAFs under both experimental conditions demonstrated a paracrine effect through secreted factors from CAFs pre-treated with FOLFOX to promote growth of CICs (**Figures 3A, B**). Interestingly, normal fibroblasts, which are not CAFs, used as control, did not increase CIC viability significantly upon FOLFOX treatment relative to untreated cells (**Figure 3A**). In agreement with the co-culture data, FOLFOX-treated CAFs enhanced the ability of CICs to initiate tumors and increase tumor growth rates in immunocompromised mice (**Figures 3C, D**). Moreover, xenografts generated by CICs co-implanted with FOLFOX-treated CAFs displayed increased SQ tumor incidence with increased tumor size (**Figures 3C, D**) and tumor numbers, and they reduced the latency of tumor formation by CICs (**Figure 3E**).

In a further step, we evaluated the effect of FOLFOX-treated CAFs on re-transplant ability of isolated colorectal patient tumor CD44v6(+)EpCAM(+)CD133(+)ALDH1(+) cells (CD44v6(+) CICs). 2 x 10^3^ CICs alone or in combination with 6 x 10^3^ CAFs pretreated with FOLFOX were subcutaneously implanted in immunocompromised mice. As shown in **Figures 3F, G**, in the immunocompromised mice models of colorectal cancer, as many as 5 x 10^5^ patient-derived non CICs (CD44v6(-) cells), did not induce tumor formation. In contrast, 5 x 10^5^ unfractionated bulk cells or as few as 2 x 10^3^ CICs resuspended in Matrigel generated visible tumors after 3 weeks (**Figures 3F–G**). In concordance with the cell culture results, FOLFOX induced enrichment of CAFs (as shown in **Figure 1D**) increased the ability of CICs to initiate tumors in immunocompromised mice (**Figures 3F, G**). Interestingly, in spite of the higher number of CICs present in 5 x 10^5^ unfractionated bulk tumor cells, tumor formation following injection of purified CICs co-implanted with FOLFOX-treated-CAFs was faster and more efficient than tumor formation obtained with the low number of CICs (**Figures 3F, G**). To investigate whether colorectal CD44v6(+) CICs co-implanted with FOLFOX-treated-CAFs display long-term tumorigenic potential, we assessed their ability to generate tumors after serial transplantations. For this purpose, indicated cells from primary SQ tumors were transplanted into secondary mice (**Figures 3F–G**). Indeed, injected CICs with FOLFOX-treated-CAFs engrafted and generated tumors that grew rapidly compared to CICs alone and required the mouse to be sacrificed within 6 weeks after implantation. Furthermore, CICs, or CICs plus FOLFOX-treated-CAFs tumor cells obtained from secondary xenografts were subsequently transplanted into third-generation mice. Thus, the CICs, alone or CICs with the FOLFOX-treated-CAF population in CRC tumors were able to generate serial xenografts showing a virtually unlimited growth potential. Interestingly, the presence of FOLFOX-treated-CAFs showed increased tumor incidence and reduced latency of tumor formation with increased size in each serial transplantation. Thus, data in **Figures 1**–**3** demonstrate that FOLFOX-therapy activates CAFs to secrete biological components in the microenvironment that stimulate CIC maintenance. Furthermore, the response to FOLFOX by these CAFs appears to be preconditioned by the tumor microenvironment since normal fibroblasts treated with FOLFOX could not induce CIC viability to the same degree (**Figure 3**, and data not shown).

### FOLFOX Stimulated Cytokine Secretion of CAFs Maintains the Viability and Tumorigenic Potential of CICs

**Figure 4:**
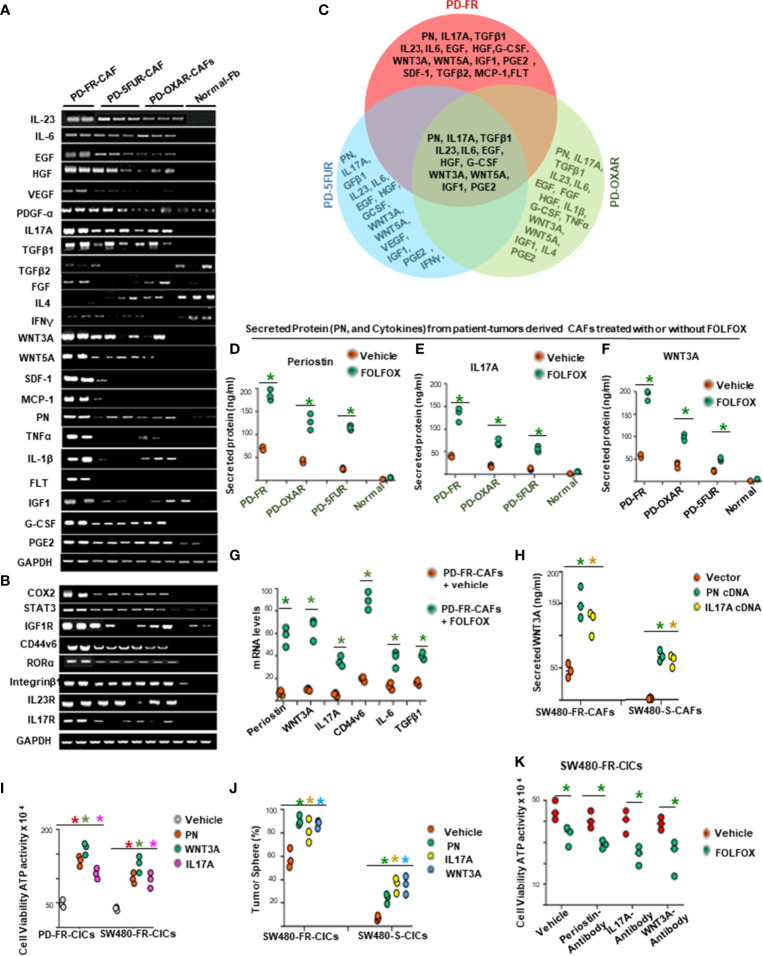
FOLFOX induces cytokine secretion in CAFs. **(A, B)**, Basal mRNA expression levels of PN and the indicated growth factors, and of growth promoting cytokines and related molecules **(A, B)**, were analyzed by semi quantitative RT-PCR in eight indicated CAFs isolated from drug resistant patient tissues and from three normal fibroblasts (Fb). GAPDH was the reference gene. **(C)**, Venn diagram shows common cytokines isolated from PD-5FUR, PD-OXAR and PD-FR from the experiment in **(A)**. **(D–F)**, CAFs derived from three patient colorectal tumor (PD)-tissues and one normal intestinal fibroblast cell line were analyzed for their PN and two dominant cytokines (IL17A and WNT3A) after FOLFOX treatment. **(G)**, QPCR analyses are shown of PN and 4 major cytokines (WNT3A, IL17A, IL6 and TGFβ1) and of CD44v6 in patient tumor CAFs treated with DMSO or FOLFOX for 72 hours. GAPDH was the reference gene. **(H)**, PN and IL17A stimulated WNT3A production in CAFs isolated from sensitive and FR tumors of SW480 were assessed by transfecting these freshly isolated CAFs by vector control, PN and IL17A expression plasmids for 72 hours. Secretion of WNT3A was measured by an ELISA assay. **(I)**, Effects of PN, IL17A and WNT3A on cell viability were assessed by using ATP Glo assay on PD-FR CICs and SW480-FR CICs. The effects were assessed in these different specimens by culturing 104 CICs with vehicle, or with 20 ng/ml of PN, IL17A or WNT 3A for 12 hours. **(J)** Percentages of colon tumor sphere formation are shown in tumor spheres isolated fromCICs of FR-resistant and sensitive cells of SW480 in the absence and presence of 50 ng/ml of either PN, or IL17A or WNT3A proteins. **(K)**, Effects of the autocrine PN, IL17A and WNT3A production in growth of tumor spheres-derived CICs (CICs) were examined by studying the effects of blockade of PN, or IL17A or WNT3A on the tumor-initiating capacity of 1 x 104 CICs. The effects were evaluated in CICs by 12-day cultures with vehicle or FOLFOX alone. Cell viabilities (# of viable cells) were measured by ATP Glo assay. Data are presented as Mean ± SD from n = 3–5 independent replicates in three independent experiments. All semi quantitative RT-PCR data are representative of three experiments. **(D–F)**, *P < 0.05 was considered significant, Secreted protein of FOLFOX treated groups compared to vehicle control group. (G), *P < 0.05, was considered significant, FOLFOX treated groups compared to vehicle control group. **(G)** *P < 0.05 was considered significant, the mRNA levels of FOLFOX treated CAFs were compared with vehicle treated CAFs. **(H)**, *P < 0.05 was considered significant, secreted WNT3A in PN, and IL17A overexpressed CAFs compared with vector control. **(I)**, *P < 0.01 was considered significant, percent cell viability stimulation with PN, WNT3A, and IL17A treatment compared with vehicle-treated cells after normalization to growth of untreated controls. **(J)**, *P < 0.05 was considered significant, percent tumor sphere stimulation with PN, WNT3A, and IL17A treatment compared with vehicle-treated CICs from S-CICs, and FR-CICs of SW480 cells after normalization to untreated controls. **(K)**, *P < 0.01 was considered significant, percent cell viability stimulation with FOLFOX treatment group compared with vehicle-treated CICs that were pre-treated with PN-, or IL17A-, and WNT3A-antibodies. Student’s t-test was used to assess the significance.

The data in **Figure 3** provide evidence that cytotoxic (FOLFOX) therapy induces remodeling of the tumor microenvironment to enrich the CAF secretome with biologically important components to support the tumorigenic potential of CICs. Therefore, using published microarray data in metastatic CRC associated fibroblasts (68), we made comparative analyses of CAF secreted cytokines, PN and some inflammatory proteins, and receptors including CD44v6, and transcription factor expressions in PD-5FUR/CAFs, PD-OXAR/CAFs and PD-FR/CAFs, as well as in normal fibroblasts (Normal-Fb) without further FOLFOX treatment (**Figures 4A, B**). **Figure 4A** (and data not shown) show several molecules that are exclusive to each CAF, and there were only 12 cytokines and matricellular protein molecules common to all three patient-tumor derived CAFs shown in the Venn diagram (**Figure 4C**). To further characterize the CAFs and compare their putative effects on CIC growth, conditioned media without serum (CM) from the three CAF and the normal fibroblast cultures were compared at baseline (without FOLFOX treatment) or after treatment with FOLFOX. Data in **Figures 4D–F** show that FOLFOX treatment significantly increased secretion of three dominant secreted factors (PN, IL17A and WNT3A) from their basal levels among the top five stromal secreted factors (PN, IL17A, WNT3A, IL-6 and TGF-β) in patient-derived PD-FR/CAFs, PD-OXAR/CAFs and PD-5FUR/CAFs (**Figure 4G**). Particularly, cytokine expression from normal fibroblasts was always lower than in CAFs basally and following FOLFOX treatment, highlighting the generally altered function of CAFs (**Figure 4D–G**, and data not shown). Regulation of these major secreted factors (PN, IL17A and WNT3A) by FOLFOX appears to occur at the level of transcription, as large increases compared to their basal levels were detectable for each of these target factors compared to IL-6 and TGF-β (**Figure 4G**). We also show that PN and IL17A were only present at ~2-7 ng/ml in sensitive cells, versus the constitutively high endogenous level (~40-59 ng/ml) observed in resistant cells (data not shown). Moreover, results of **Figures 4H, F**, and **Supplemental Figure 1A** indicate that both PN and IL17A induced WNT3A mRNA expression and secretion. Therefore, in the present study, we focused on PN, WNT3A and IL17A. Overall, these data strongly suggest that CAFs respond to FOLFOX treatment by secreting paracrine signaling factors, including matricellular proteins, cytokines, and growth factors.

In order to determine the involvement of these stromal-factors on CIC maintenance, we analyzed the autocrine production of the three stromal-secreted factors in PD-FR/CICs that were previously treated with vehicle or FOLFOX in the absence of CAFS. The results showed insignificant or only moderate levels of autocrine productions of PN, WNT3A and IL17A by CICs compared to their levels in CAFs (**Supplementary Figure 1B**, versus **Figures 4D–F**), and treatment with FOLFOX greatly increased the gene expressions and secreted proteins by these three paracrine factors (**Figures 4H**, and **Supplementary Figure 1A**). Moreover, each of these three stromal-secreted factors (PN, WNT3A or IL17A) directly augmented CICs viability in the absence of CAFs (ATP activity, **Figure 4I**) with reduced apoptosis (apoptosis data not shown). Further, these three CAF-secreted factors stimulated tumorigenic potential of CICs as measured by tumor sphere formation in CICs from sensitive and FR tumors of SW480 (**Figure 4J**). Note that the basal tumorigenic activity of SW480-FR-CICs was much higher compared to SW480-S-CICs, and treatment with each of the three stromal-secreted factors (PN, WNT3A and IL17A) increased tumor sphere formation ability relative to vehicle treated CICs (**Figure 4J**). These results provide evidence that these CAF-derived factors might directly regulate CIC maintenance. Moreover, both PN and IL17A increase WNT3A secretion (as in **Figures 4H** and **Supplementary Figure 1A**) indicating that WNT3A signaling may have a crucial role in tumor cellular hierarchy. To further elucidate the possible role for autocrine effects of PN, IL17A and WNT3A on CICs, we investigated the effects of blocking each of these stromal factors on the viability of CICs (measured by Cell Titer-Glo assay) and on the tumorigenic potential of CICs (measured by quantitation of tumor spheres) in the absence of CAFs using a blocking antibody for each of the stromal factors (**Figures 4K** and **Supplementary Figure 1C**). Only moderate levels of viability and tumorigenic potential were noted as a result of PN, or IL17A or WNT3A blocking antibodies in CICs (**Figuress 4K** and **Supplementary Figure 1C**), suggesting that CAF-derived PN, IL17A and WNT3A (**Figures 4D–F**) have a major affect compared to autocrine production of these factors in CICs. Further, blocking IL17A, PN or WNT3A only reduced tumor sphere formation in CICs isolated from resistant cells, while having no effect in CICs derived from sensitive cells (**Supplementary Figure 1C**) indicating that PN, WNT3A and IL17A induced tumorigenic activity is confined to the role of increased CAFs after FOLFOX treatment (as seen in **Figure 1D**).

### PN and IL17A Can Contribute to CIC Maintenance Through WNT3A-CD44v6 Signaling

**Figure 5:**
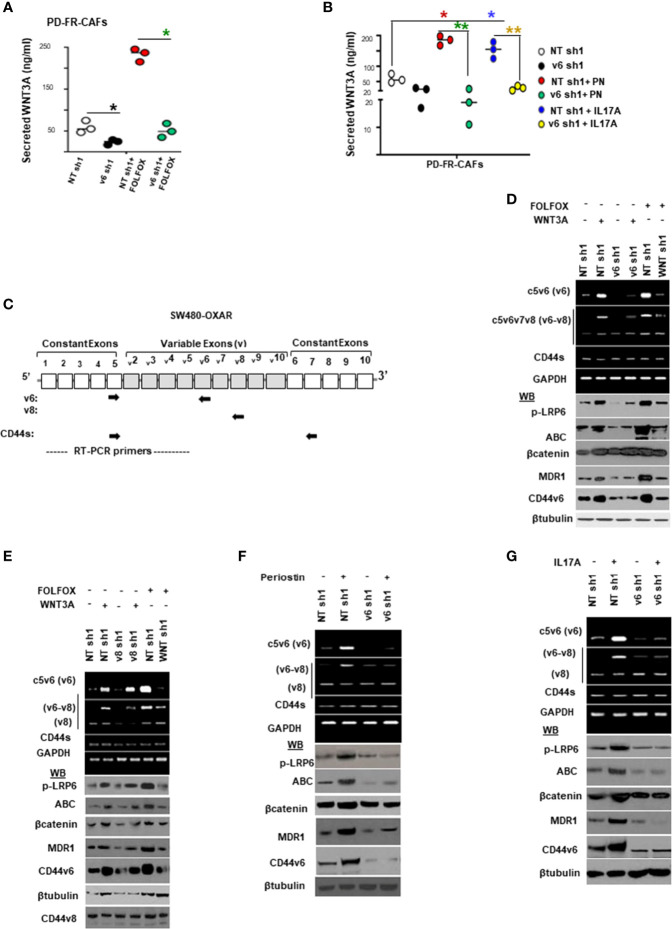
FOLFOX induces CD44v6 expression, which is critically regulated by the WNT pathway stimulated by PN and/or IL17A. **(A)**, Confirmation of involvement of CD44v6 expression in regulating WNT3A production was assessed in PD-FR CAFs by examining the effects of blockade of CD44v6 using specific shRNAs on 10^4^ SW480-FR/CAFs in the absence and presence of FOLFOX. The effects were evaluated by measuring secreted WNT3A in cultures by ELISA. **(B)**, Involvement of CD44v6 in regulating PN and IL17A induced WNT3A production was assessed in PD-FR CAFs by examining the effects of blockade of CD44v6 using specific shRNAs in the absence and presence of PN or IL17A. The effects were evaluated by measuring secreted WNT3A in cultures by ELISA. **(C)**, Schematic illustration of the CD44 gene is shown. Both constitutive **(C)** and variable (v) exons are represented. The PCR primers used to amplify CD44 variable (v) and standard (s) isoforms are shown as black arrows. The primers for both the v6 and standard isoforms (CD44s) predominantly generate one PCR product [C5v6 (v6) for CD44v6, and C5C7 for CD44s], whereas the primers for the v8 variants amplify two splice variants C5v6v7v8 (v6-v8) and C5v8 (v8). These PCR products are depicted in panels **(D–G)** experiments. The primers for both the v6 and standard isoforms predominantly generate one PCR product [c5v6 (v6)], whereas the primers for the v8 variants amplify two splice variants C5v6v7v8 (v6-v8), and C5v8 (v8). **(D, E)**, WNT3A shRNA, or CD44v6 shRNA1 **(D)**, but not CD44v8 shRNA1 **(E)** Upper panels: Semi-quantitative RT-PCR analyses are shown for CD44 variants in SW480-OXAR SQ tumor cells transfected with NT sh, or v6 sh1 or WNT3A sh1 followed by WNT3A or FOLFOX stimulation for 12 hours. Lower panels: Western blot analyses are shown for p-LRP6, active β−catenin, total β−catenin, MDR1, CD44v6 or β-tubulin. **(E)**, Upper panels: Semi-quantitative RT-PCR analyses are shown for CD44 variants in SW480-OXAR SQ tumor cells transfected with NT sh, or v8sh1, or WNT3A sh1 followed by WNT3A or FOLFOX stimulation for 12 hours. Lower panels: Western blot analyses are shown for p-LRP6, active β−catenin, total β−catenin, MDR1, CD44v6 or β-tubulin. **(F, G)**, Upper panels: Semi-quantitative RT-PCR analyses are shown for CD44 variants in SW480-OXAR SQ tumor cells transfected with NT sh or v6 sh1 followed by PN or IL17A stimulation for 12 hours. Lower panels: Western blot analyses are shown for p-LRP6 (S1490), active β−catenin, total β−catenin, MDR1, CD44v6 or β-tubulin. Data in A, B, represent means ± SD; n = 3–6 independent replicates in three independent experiments. **(A)**, *p < 0.05 was considered significant, inhibition of WNT3A secretion in v6 shRNA1 groups compared to NT shRNA1 controls. **(B)**, *p < 0.05, **p < 0.01 were considered significant, Inhibition of WNT3A secretion in v6 shRNA1 +PN and v6 shRNA1 +IL17A groups compared to NT shRNA1 controls. All Western blots and semi quantitative RT-PCR data are representative of three experiments.

Given that CAFs derived from resistant tumor cells (PD-5FUR, PD-OXAR and PD-FR CAFs) also express significant levels of CD44v6 compared to its absence in normal-fibroblasts (as seen in **Figure 4B**), we determined if CD44v6 induces WNT3A activation in CAFs since it regulates WNT3A/β-catenin signaling in CICs (48). Thus, first, WNT3A expression was analyzed in FOLFOX treated PD-FR CAFs in which CD44v6 was knocked down, and the results show that CD44v6 regulates WNT3A production in FOLFOX treated PD-FR/CAFs (**Figure 5A**). Furthermore, this regulation of CD44v6 on WNT3A is through IL17A and PN in FR-tumor cell derived CAFs (**Figure 5B**), and this regulation of CD44v6 on WNT3A, PN and IL17A was not found in sensitive S-tumor cell derived CAFs (data not shown). Second, to investigate whether CD44v6 variants are important for *β*-catenin-MDR1 signaling, we examined the effects of WNT3A and FOLFOX on activation of CD44v6 splicing and *β*-catenin-MDR1 signaling in oxaliplatin resistant SW480-OXAR/SQ tumor cells. To detect the effects on CD44v6 and CD44v8, exon specific PCRs were examined using two different 3’ primers paired to either the v6 or v8 exon respectively, and to a primer to the 5’ constitutive exon 5 (c5) (as shown in schematic diagram **Figure 5C**; primers are shown in **Table 2**). As shown in **Figure 5D**, following WNT3A or FOLFOX addition, the expression of CD44v6 variants increased significantly, whereas the expressions of the CD44v8 isoform or the standard CD44s isoform were not increased. Along with the CD44v6-containing isoform, the activation of *β*-catenin modulators, including p-LRP6, active *β*-catenin and MDR1 expressions were upregulated. Knocking down CD44v6 or WNT3A inhibited the induction of CD44v6 and WNT/*β*-catenin-MDR1 signaling (**Figure 5D**). The residual expressions of CD44 variants after WNT3A knockdown suggests that other signaling cascades, in addition to WNT/β-catenin, may also regulate v6 splicing of CD44 (**Figure 5D**). However, the levels of the v8 variant and of CD44s (with no variant exon) were almost identical after treatment with either v6 shRNA expressing cells (**Figure 5D**) or to the levels of control shRNA (NT-shRNA) treated cells. Treating cells with CD44v8shRNA and either WNT3A or FOLFOX inhibited the up-regulation of all the examined CD44v8-containing variants, but not the CD44v6 variants, and not the activation of p-LRP6, active-*β*-catenin and MDR1 expressions (**Figure 5E**). WNT3A, or FOLFOX stimulated isoforms containing v6 were silenced by WNT3A shRNA, or CD44v6 shRNA (**Figure 5D**), but not by CD44v8 shRNA (**Figure 5E**). This indicates that CD44v6 signaling, but not CD44v8 signaling, is required for WNT3A-, or FOLFOX-induced WNT/*β*-catenin-MDR1 signaling (**Figure 5E**). The v6–v8 variant amplified in the same experimental RT-PCR analysis maintained down-regulated levels (42 ± 5.0% of NT shRNA levels) because this variant contained the v6 exon targeted by v6 shRNA1 (**Figure 5E**). These results (**Figures 5D, E**) show that the CD44v6 containing isoform and CD44v6/LRP6/*β*-catenin/MDR1 signaling triggered by stromal secreted WNT3A might be important for drug resistance in CICs.

**Figure 6:**
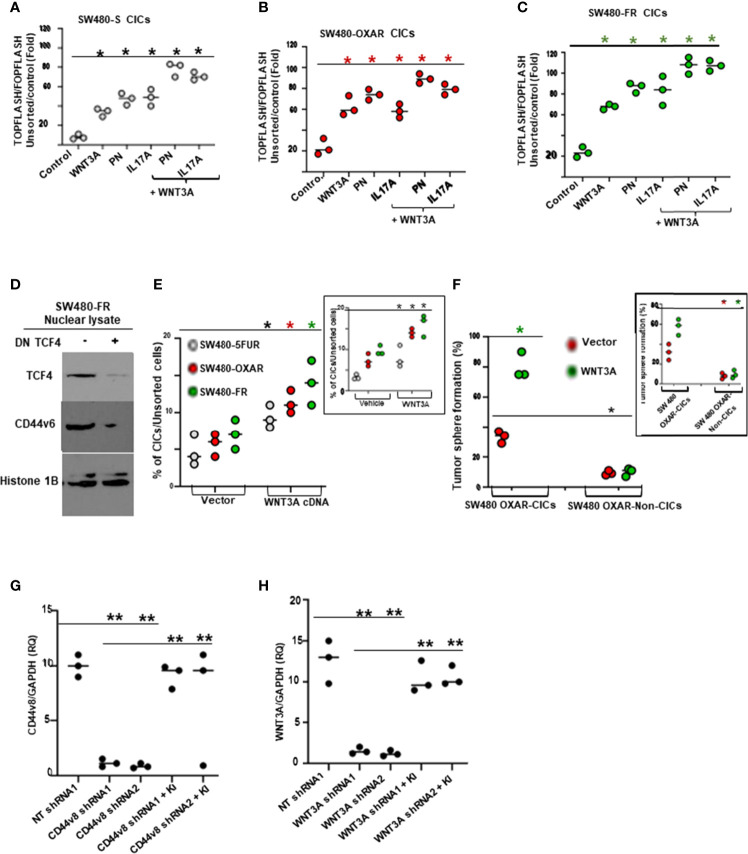
FOLFOX induces b-catenin/TCF4 luciferase activity and tumor sphere formation in CICs stimulated by PN and/or IL17A, and TCF4 regulates CD44v6 expression. **(A–C)**, WNT reporter (TOPFLASH/FOPFLASH) activities were determined in SW480-S **(H)**, SW480-OXAR **(I)** and SW480-FR **(J)** CICs in response to culturing with vehicle (Control), or with PN, or IL17A or WNT3A alone (20 ng/ml), or in combination PN + WNT3A or IL17A + WNT3A. **(D)**, Dominant negative TCF4 mediated down regulation of TCF4 inhibits CD44v6 expression in nuclear lysates of SW480-FR SQ tumor cells. **(E)**, Flow cytometry analyses of percent enrichment of CICs are shown for unsorted cells from SW480-5FUR/SQ, SW480-OXAR/SQ and SW480-FR/SQ cells overexpressing WNT3A or vector control. **(F)**, Percentages of colon tumor sphere formation were measured in a sphere-formation assay for CICs and Non-CICs in SW480-OXAR SQ tumor cells overexpressing WNT3A or vector control. **(G,H)**, The effects of shRNA-mediated knockdown of CD44v8 and WNT3A using specific shRNAs in SW480-FR cells on the expressions of CD44v8 mRNA and WNT3A mRNA, as determined by real-time PCR (at 24 h; RQ, relative quantification) were done by the indicated shRNA mediated knockdown and the corresponding knock-in (KI) gene transfections as described in Methods. Target mRNAs were analyzed by QRT-PCR analysis (GAPDH, internal control). Data in **A–C, E–H**, represent means ± SD; n = 3–6 independent replicates in three independent experiments; **(A–C)**, *p < 0.05 was considered significant, stimulation of TOPFLASH/ FOPFLASH activities in treatment groups compared to control groups. **(D)**, Western blot data are representative of three experiments. **(E)**, *p < 0.05 was considered significant, stimulation of % of CICs by WNT3A over expression or exogenously added WNT3A group (inset) compared to respective control groups. , **(F)**, *p < 0.05 was considered significant, stimulation of tumor sphere formation in WNT3A over expression or exogenously added WNT3A group (inset) compared to respective control groups. **(G, H)**, **p < 0.01 was considered significant, Fold inhibition of CD44v8 and WNT3A mRNA in shRNAs overexpressing groups compared to corresponding NT shRNA groups; Fold restoration of CD44v8 and WNT3A mRNA in corresponding shRNA + Knock-in (KI) groups compared to corresponding shRNA groups. Student’s t-test was used to assess the significance.

To gain insight into how PN and IL17A are related to the mechanisms that control viability of CICs and tumorigenic potentiality, we determined whether PN and IL17A coordinated with WNT to activate WNT-CD44v6-MDR1 signaling as described in our companion paper (48). Thus, we measured expressions of CD44 variants by RT-PCR, and levels of p-LRP6 (S1490), active *β*-catenin and MDR1 by WB after PN and IL17A stimulation in SW480-FR cells. As shown in **Figures 5F–G**, following addition of PN (**Figure 5F**) or IL17A (**Figure 5G**), expressions of CD44v6 and v6-containing variants increased, while the standard form (CD44s) remained unchanged. Activation of LRP6, active *β*-catenin and MDR1 also increased following PN or IL17A treatment, which was abrogated following v6 shRNA1 transfection. The expressions of variants containing the v6 exon were increased by greater than three (3.7 ± 0.54) and six (6 ± 0.53) fold respectively. Increases of 1.8 ± 0.33 and 2.6 ± 0.53 fold were observed for the v6–8 for PN and IL17A addition respectively (**Figures 5F, G**). Expression of CD44v6shRNA1 in PN- and IL17A-treated cells inhibited the up-regulation of all the CD44v6-containing variants. In addition, the interaction between either PN or IL17A with CD44v6-downstream signaling activities were measured by LRP6 phosphorylation, and by expressions of active β-catenin and MDR1 proteins (**Figures 5F, G**). TOPFlash carries TCF-binding sites, which are directly activated by the TCF4/β-catenin complex (69, 70). The data in **Figures 6A–C** indicate that PN, IL17A or WNT3A treatment in CICs from sensitive and chemo-resistant colorectal tumors induces the TOPFlash reporter activity. In each case each of these paracrine factors increases the reporter activity from their basal activity levels, and the combined treatment further stimulates the transactivation of the TOPFlash reporter (**Figures 6A–C**).

Given that TCF4 binding sites are present in the CD44v6 promoter sequence as described in our companion paper (48), we determined whether FOLFOX induced increased expression of CD44v6 depends on TCF4. Thus, a dominant-negative TCF-DN was co-transfected in resistant SW480-FR cells, and their CD44v6 expression in nuclear fractions was strongly inhibited (**Figure 6D**). Furthermore, WNT3A signaling activity is increased in the CD44v6(+)CIC population isolated from SW480-5FUR, SW480-OXAR and SW480-FR SQ tumor cells overexpressing WNT3A, as analyzed by FACS (**Figure 6E**). Flow cytometry analysis showed that following the enrichment of CICs in WNT3A transfectants, a majority of CICs were present in sorted SW480-FR/SQ tumor cells expressing constitutively high WNT3A activity (**Figure 6E**), and this WNT3A activity is required for maintenance of the CIC immunophenotype (CD44v6 [high+]) by promoting tumor sphere formation in CICs (**Figure 6F**). Validations of CD44v6 shRNAs, CD44v8 shRNAs and WNT3A shRNAs were done following our previously published paper (31, 49) and are shown in **Figures 2G**, **6G**, and **6H**. Thus, PN, WNT3A and IL17A act as CIC niche components that can promote CIC maintenance and expansion by augmenting WNT-CD44v6-*β*-catenin-MDR1-signaling. This can trigger therapeutic resistance in CICs and their ability to generate virtually unlimited growth potential in serial xenografts established by injecting CICs in combination with CAFs subcutaneously in

immunocompromised mice (as seen in **Figure 3F–G**).

### PN and IL17A Induce Association of Nuclear *β*-catenin/TCF4 With CD44v6 and MDR1 to Modulate Drug Resistance

**Figure 7:**
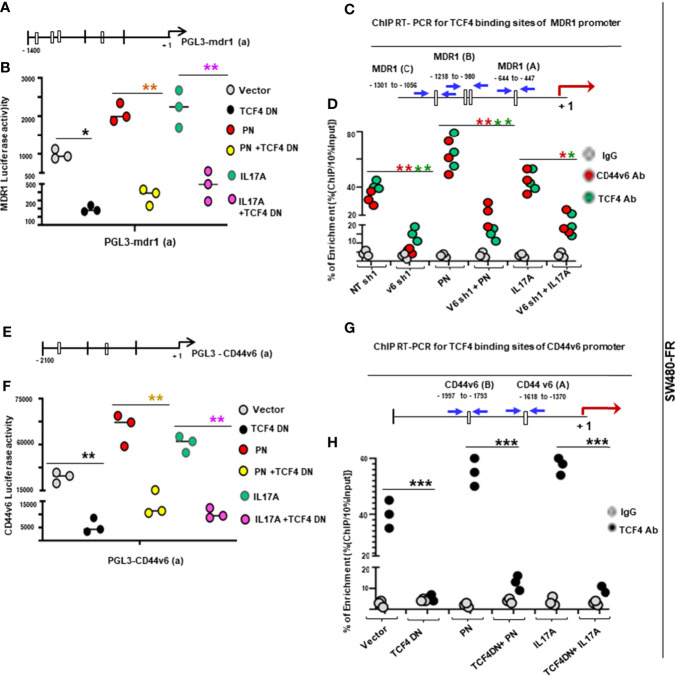
PN and IL17A induce association of nuclear β-catenin/TCF4 with CD44v6 and MDR1 to modulate drug resistance. **(A, B)**, PN and IL17A induced MDR1 promoter luciferase activities were measured in SW480-FR cells using the indicated pGL3-mdr1 (a) reporter containing TCF4 binding sites (CTTTGA). **(A)**, The scheme shows the pGL3-mdr1 (a) reporter constructs with TCF binding sites. **(B)**, MDR1 luciferase activity reporter assays are shown for SW480-FR cells overexpressing a dominant negative TCF4-DN construct for 48 hours followed by co-transfection with or without PN or IL17A expression vectors. **(C)**, The sketch map shows the predicted TCF4 binding sites (CTTTGA) within the indicated MDR1 promoter (blue arrows). The transcriptional start site (red arrow) was at “+1”, and ATG is at the translation start site. The putative TCF4 binding sites (MDR1 (A), MDR1 (B) and MDR1 (C) are shown, and their locations are labeled. **(D)**, ChIP PCR primers, designated for MDR1 **(A)** as shown in **(C)**, were used for amplification of the potential TCF4 binding sites of the MDR1 gene. ChIP assays were done using either anti-CD44v6, anti β-catenin or irrelevant IgG antibody as negative control in SW480-FR cells overexpressing specific shRNAs against CD44v6 or Non-targeted shRNA (NT-sh) with or without co-transfection with PN or IL17A constructs. Input: total genomic DNA was used as control for the PCR. **(E, F)**, PN and IL17A induced CD44v6 promoter luciferase activities were measured in SW480-FR cells using the indicated pGL3-CD44v6 (a) reporter containing TCF4 binding sites. **(E)**, The sketch map of predicted TCF4 binding sites (CTTTGA) within the CD44v6 promoter luciferase construct (CD44v6 [a]) is shown. **(F)**, CD44v6 luciferase activity reporter assays are shown for SW480-FR cells overexpressing dominant negative (DN) TCF4 for 48 hours followed by co-transfection with or without PN or IL17A expression vectors. **(G, H)**, PN or IL17A induced CD44v6 gene expression regulated by TCF4 in SW480-FR cells is shown. **(G)**, The sketch map shows the predicted TCF4 binding sites (CTTTGA) within the indicated CD44v6 promoter (blue arrows). The transcriptional start site was at “+1”, and ATG is at the translation start site (red arrow). The putative TCF4 binding sites [CD44v6 **(A)**] and CD44v6 **(B)** are shown, and their locations are labeled. **(H)**, ChIP PCR primers, designated for CD44v6 **(A)** as shown in **(G)**, were used for amplification of the potential TCF4 binding sites of the CD44v6 gene. ChIP assays were done using either anti-TCF4 or irrelevant IgG antibody as negative control in SW480-FR cells overexpressing a dominant negative TCF4-DN construct for 48 hours followed by co-transfection with or without PN or IL17A expression vectors. Data presented in **(B, D, F, H** are Mean ± SD.; n = 3–5 independent replicates in three independent experiments; **(B)**, *, P < 0.05, **, P < 0.01 were considered significant, Inhibition in MDR1 luciferase activity in TCF4 DN, or PN + TCF4 DN, or IL17A + TCF4 DN overexpressed groups compared to vector control group. **(D)**, *, P < 0.05, **, P < 0.01 were considered significant, Inhibition of percent of enrichment of MDR1 mRNA in v6 shRNA1 (v6 sh1), v6 sh1+PN, v6 sh1+IL17A groups compared with , NT sh1, or PN, or IL17A overexpressed groups. **(E)**, *, P < 0.05, **, P < 0.01 were considered significant, Inhibition in CD44 luciferase activity in TCF4 DN, or PN + TCF4 DN, or IL17A + TCF4 DN overexpressed groups compared to vector control group. **(F)**, *, P < 0.05, **, P < 0.01, ***, P < 0.001 were considered significant, Inhibition of percent of enrichment of CD44v6 transcripts in TCF4 DN, or PN + TCF4 DN, or IL17A + TCF4 DN overexpressed groups compared to vector control group. Student’s t-test was used to assess the significance.

PN and IL17A mediation of a WNT3A/β-catenin pathway regulates CD44v6 expression and vice versa (as seen in **Figures 5A,B,D–G**), and enriches CD44v6 in CICs (**Figures 6D**). In searching for a possible linkage between PN and IL17A with WNT/*β*-catenin signaling to regulate CD44v6 expression and CIC survival/self-renewal, we analyzed transient transfection assays using SW480-FR cells with constructs containing TCF binding sites within the MDR1 promoter cloned into a luciferase reporter plasmid (see the schematic model of MDR1 luciferase reporter in **Figure 7A**). This construct was transfected with or without manipulations of TCF4 knockdown followed by either PN or IL17A co-transfection, and luciferase activities were measured for the PGL3-mdr1 constructs (**Figure 7B**). The results showed an ~8-fold decrease of basal PGL3-mdr1 luciferase activity by knocking down TCF4 (**Figure 7B**). In a further step, to determine the linkage between PN and IL17A with WNT/*β*-catenin signaling to regulate CD44v6 gene expression, we analyzed transient transfection assays using SW480-FR cells with constructs containing TCF binding sites within the MDR1 promoter cloned into a luciferase reporter plasmid. The cells were transfected with or without manipulations of TCF4 knock down followed by either PN or IL17A co-transfection, and luciferase activities were measured for the PGL3-mdr1 constructs (scheme in **Figure 7A**). The results showed that either PN or IL17A overexpression increases this promoter activity ~3 fold and ~3.5 fold respectively (**Figure 7B**). In contrast, the MDR1 promoter luciferase constructs negatively responded to co-transfection of dominant-negative TCF4-DN in cells pre-transfected with PN or IL17A constructs (**Figure 7B**). These reductions provide evidence that TCF promoter binding and activation of MDR1 is mediated through both PN and IL17A stimulated TCF4 in the nucleus. In agreement with these results, ChIP assays were done using CD44v6- immunoprecipitated (IP) DNA, and TCF4-IP’d DNA, and input DNAs were amplified using primers covering the indicated TCF4 binding sites (see the schematic model of MDR1 promoter containing TCF4 binding sites in **Figure 7C**). ChIP assays (**Figure 7D**) showed robust binding of *β*-catenin/TCF4 to MDR1 sites in the SW480-FR cells. Results also showed a robust binding activity for CD44v6 to the DNA fragment where TCF4 binds (**Figure 7D**). Knockdown of CD44v6 reduced endogenous *β*-catenin/TCF4 binding to the *MDR1* promoter in response to PN and IL17A in SW480-FR cells (**Figure 7D**), thus validating the results from the luciferase reporter assay (**Figure 7B**) that CD44v6 and β-catenin co-regulate MDR1 expression in response to PN and IL17A in FOLFOX resistant cells. ChIP assays showed that at basal level, *β*-catenin/TCF4 bound weakly to TCF binding sites of the *MDR1* promoter in sensitive cells compared to robust binding activity in resistant cells (data not shown). These data provide evidence that in the presence of CD44v6, TCF4-mediated transactivation of *MDR1* was up-regulated from basal activity to CD44v6-regulated activity *via* TCF4 in response to PN and IL17A. When stabilized *β*-catenin enters the nucleus, it interacts with transcriptional regulators, including TCF4, which leads to WNT/*β*-catenin responsive gene expression (69). Thus, the results in **Figure 7D** indicate that PN and/or IL17A regulate β-catenin/TCF4 binding to a *MDR1* promoter in a CD44v6-regulated manner in FOLFOX resistant cells.

Several putative TCF binding sites were located 2 kb upstream of the transcriptional start site of the CD44v6 gene (see the schematic model of CD44v6 luciferase reporter in **Figure 7E**). A CD44v6 luciferase assay was used to directly examine the interaction between PN and IL17A induced *β*-catenin/TCF4 and the CD44v6 promoter. The luciferase activities in SW480-FR cells transfected with dominant negative TCF4-DN were significantly lower than vector group, while PN and IL17A overexpression significantly increased the luciferase activity (**Figure 7F**). Further, this result also indicates that PN and IL17A induced CD44v6 promoter activity requires WNT/TCF4 binding to the CD44v6 promoter. This provides evidence that PN and IL17A stimulated the *β*-catenin/TCF4-mediated increase in CD44v6 transcription activity. To further validate these results, conventional ChIP analyses were done, and they provided direct evidence for the ability of PN and IL17A to stimulate TCF4 to bind to the promoter of CD44v6 (see the scheme in **Figure 7G**, and CD44v6 transcription activity in **Figure 7H**). The data in **Figure 7** indicate that PN and IL17A promote both CD44v6 and MDR1 gene expressions through a *β*-catenin/TCF4 pathway. Overall, the above results (**Figure 7**) indicate that FOLFOX treatment mediates PN and IL17A, which recruit a WNT3A pathway that promotes CD44v6 expression (as seen in **Figures 5**, and in MDR1 and CD44v6 transcription activities in **Figure 7**), and CD44v6 regulates WNT3A production (as seen in **Figures 5A, B** and β-catenin signaling through PN and IL17A (as seen in **Figures 5D–G**) in response to FOLFOX treatment. Taken together our results provide evidence for a positive feed-back loop between CD44v6 and β-catenin/TCF4 that activates MDR1 gene expression and CD44v6 splicing, and mediates FOLFOX resistance (as seen in **Figures 2F**, **5** and **7**).

### Stromal Secreted Factors Can Maintain CIC Growth and Tumorigenic Function

**Figure 8:**
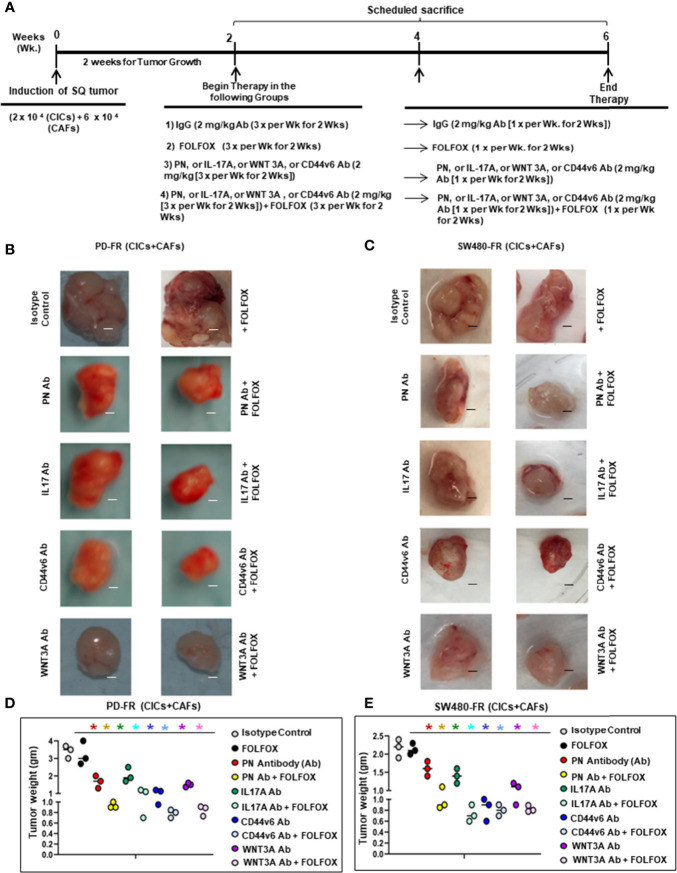
FOLFOX induced PN, WNT3A and IL17A signaling regulates CIC growth *in vivo*. **(A),** Timeline is shown for antibody treatments with and without FOLFOX in xenograft tumors implanted by a mixture of CICs plus CAFs from PD-FR human tissues **(B, D)** and from SW480-FR xenograft tumor cells **(C, E)**. The dependences of CICs on IL17A, WNT3A, PN and CD44v6 were evaluated *in vivo*. 2 x10^4^ CICs and 6 x 10^4^ CAFs were injected into mice. When tumors reached to approximately 0.3 cm^3^ in volume, treatments were initiated. Detailed treatment procedure is in Methods. Mice were weighed, and the tumor volumes were measured every other day for 4 weeks. **(B, C),** Representative tumors from three experiments are shown from mice treated as indicated. **(D, E),** Tumor weights from the experiments in **(B, C)** for each tumor set at the end of treatment after 4 weeks are shown. Data presented in **(D, E)** are Mean ± SD.; n = 5 mice/group independent replicates in three independent experiments; **(B)**, *, P < 0.05 were considered significant, inhibition in tumor growth represented by tumor weight and tumor volume in treatment groups compared to IgG control groups. ANOVA, followed by Bonferroni’s post-hoc test was used to assess the significance. Scale bars, 1 cm.

To address the contributions of PN, WNT3A, IL17A or CD44v6 in the *in vivo* SQ growth of CICs, a neutralizing antibody for each of these factors was used in combination with 1 x FOLFOX (schematic model detailing the experimental procedures (**Figure 8A**) used in the experiment of **Figure 8B**). Tumors were established by injecting CICs in combination with CAFs subcutaneously in immunocompromised mice. Administration of neutralizing antibodies for PN, or WNT3A, or IL17A, or CD44v6 increased the efficacy of FOLFOX-chemotherapy on reducing tumor growth (**Figures 8B–E**). These results confirm that FOLFOX resistance depends primarily on secreted factors including PN, WNT3A and/or IL17A in CAFs, as well as on CD44v6 in both CICs and CAFs. These data provide evidence that the central roles of PN, WNT3A and/or IL17A are to serve more as paracrine signals to have crucial roles in driving FOLFOX resistance in CICs and in promoting tumor growth. Together our data support a pattern in which FOLFOX-therapy stimulates CAFs and activates CIC maintenance (as seen in **Figure 1D**, and **Figure 3**) in the microenvironment to stimulate paracrine signals (as seen in **Figure 4**) for induction of CIC growth (as seen in **Figures 3C–G**) and SQ tumor growth when implanted with CAFs and CICs (**Figures 8B–E**).

### Tissue Specific Inhibition of CD44v6 *In Vivo* Inhibits MDR1 Expression and CRC Progression by Retaining FOLFOX Sensitivity

There is no small molecule inhibitor for CD44v6 commercially available. For genetic targeting of CD44v6, we tested the effect of pSico-CD44v6shRNA plus pFabpl-Cre by intraperitoneal treatment with nanoparticle delivery systems engineered to express CD44v6shRNA in the SW480-FR/CICs plus PD-FR/CAFS implanted xenograft tumors in immunocompromised mice. The purpose was to transactivate a conditionally silenced pSico- plasmid with a CD44v6 shRNA oligonucleotide (pSico-CD44v6shRNA) by Cre-recombinase that is produced in response to an intestine/colon tissue-specific pFabpl promoter. The principle is as follows. The recombinase, produced under the influence of a tissue-specific promoter in the cells, will eliminate the (CMV-EGFP)-cassette from U6-(CMV-EGFP)^flox/flox^-CD44v6 shRNA from the pSico-CD44v6shRNA, and then the U6 promoter will induce synthesis of CD44v6 shRNA. Normal cells in the intestine/colon will not be affected because they rely mostly on the standard CD44s expression, which does not have any variant exons of CD44. Even if cells in other organs express CD44v6shRNA, knockdown of CD44v6 will not be produced due to lack of response to the tissue-specific promoter. Unused plasmids in other organs will be progressively destroyed by cytoplasmic nucleases, and the nanoparticles will be cleared because PEG and PEI are biodegradable, thus avoiding any toxicity problem (31, 49), It has been shown that Transferrin (Tf)-receptors are highly expressed in colon tumors (59).

**Figure 9:**
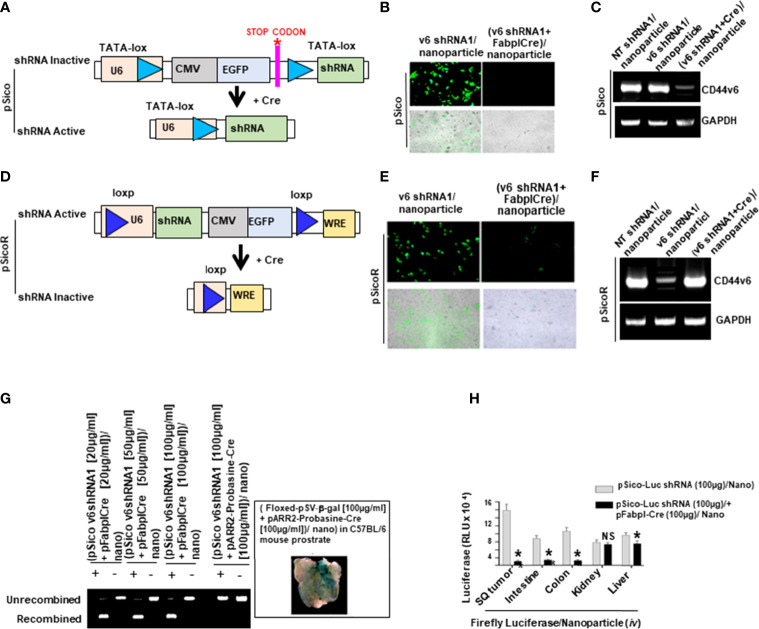
Confirming the inducible CRE system used tissue specific knockdown of CD44v6 to inhibit subcutaneous (SQ) tumor growth. **(A–D)**, Schematic representations of pSico, and pSicoR after Cre-mediated recombination to synthesize active shRNA are shown. **(B–E)**, SW480-FR/SQ tumor cells from the transfection of the cells with (pSico-v6shRNA1 plus the pFabpl-Cre)/transferrin (Tf)-PEG-PEI) (Nanoparticles [Nano]) and with the (pSicoR-v6shRNA1 plus pFabpl-Cre)/Nano and were analyzed by epifluorescence microscopy to detect GFP. Similar cell densities and identical exposure times were used for all images. (See Materials and Methods for details in nanoparticles). **(C, F)**, Total RNAs extracted from the transfected cells were analyzed for CD44v6 and GAPDH mRNAs by semi-quantitative PCR. **(G)**, SW480-FR cells were transfected with pSico v6 shRNA plus Fabpl-Cre/Nano, or with pSicoR v6shRNA plus Fabpl-Cre/Nano. PCR detection is shown for Cre-mediated recombination of pSico-CD44v6 shRNA in tumor Genomic DNAs extracted from mice 4 days after shRNA plus indicated Cre/Nano treatments to xenograft tumors induced by SW480-FR cells. (# of mice/experiment = 5; # of experiments = 2). Note that pSico v6 shRNA plus Probasin Cre/Nano did not have any effect on colon tumors. Inset shows the functionality of Floxed-pSVβ-galactoside/ plus pARR2-Probasin Cre/nanoparticles in the prostate gland. **(H)**, Validation for tissue specific delivery of shRNA/nanoparticles was done by verifying distributions of shRNA against the firefly luciferase gene (pSico-Firefly luciferase shRNA plus pFabpl-Cre)/Nano, and are shown in various organs of C57/Bl mice (n = 4), which were previously injected with reporter plasmids expressing firefly luciferase and renilla luciferase (i.p.). The luciferase activities of the tumor extracts and the indicated tissues were measured. Note that luciferase-shRNA is active in colon tumors and in normal colon and intestine tissues due to the functionality of pFabpl-Cre. Data presented are Mean ± SD.; n = 5 mice/group independent replicates in two **(G)** and three **(H)** independent experiments. All semi quantitative RT-PCR and Epifluorescence microscopy data are representative of three experiments. **(H)**, *, P < 0.05 was considered significant, inhibition in luciferase activity in (pSico-Luc sh (100μg) + pFabpl-Cre (100μg))/ Nano compared to pSico-Luciferase shRNA (100μg)/Nano group. Student’s t-test was used to assess the significance in **(H)**.

First, to assess the contributions of CD44v6 in colorectal CICs and CAFs implanted SQ tumors, we generated pSico-CD44v6shRNA plus pFabpl-Cre plasmids and encapsulated them in a Tf-polyethylene glycol (PEG)-polyethylene imine (PEI) nanoparticles following our previous study (59). The pSico-CD44v6shRNA was used in *in vivo* experiments in the presence of Fabpl-Cre to constitutively silence endogenous CD44v6 (schematic representation of pSico is shown in **Figure 9A**). The same CD44v6shRNA sequence was cloned in a pSicoR vector (**Figure 9D** schematic representation of pSicoR) and was used to constitutively silence endogenous CD44v6 in the absence of Fabpl-Cre in *in vitro* experiments. As a control, non targeted (NT) shRNA was also cloned in these vectors. Second, before applying our pSico-CD44v6shRNA1 plus Fabpl-Cre in *in vivo* experiments, we validated our Cre-regulated v6-shRNA1/nanoparticles in SW480-FR cells, which were transfected with either pSico-CD44v6shRNA1 with or without Fabpl-Cre containing nanoparticles. High-efficiency transduction by either vector was achieved as indicated by uniform GFP expression (**Figure 9B** for pSico-CD44v6shRNA1 and **Figure 9E** for pSicoR-CD44v6shRNA1 with or without Fabpl-Cre nanoparticles). Validation of the v6shRNA1 was done in **Figure 2G** following our previously published papers (31, 49). CD44v6shRNA1 expression by this vector was functionally consistent in inducing large reductions of CD44v6 gene expression, which was Cre-dependent (**Figures 9B, C** for pSico-CD44v6shRNA1 and **Figures 9E, F** for pSicoR-CD44v6shRNA1 with or without Fabpl-Cre nanoparticles). The results of **Figures 9A–H** validate the use of Fabpl-Cre- and pSico-CD44v6shRNA1, and pSicoR-CD44v6shRNA1 nanoparticles for silencing CD44v6 expression in *in vitro* and in *in vivo* experiments.

Further we validated the inclusion of a pFabpl-Cre plasmid in our conditional silencing of CD44v6 in *in vivo* tumors derived from CICs plus CAFs isolated from SW480-FR tumor cells. As shown in **Figure 9G**, shRNA knockdown of CD44v6 in CICs plus CAFs implanted to form SW480-FR/SQ tumors depended on the inclusion of a pFabpl-Cre plasmid and induced nearly complete recombination due to loss of GFP. In contrast a pARR2-Probasin-Cre plasmid, which is specific for prostate tissue, could not induce a recombination of the pSico-CD44v6-shRNA plasmid and did not show any effect in colon tissue. The inset (**Figure 9G**) shows that pARR2-Probasin Cre releases β-galactosidase from a conditional β-gal plasmid in the mouse prostate, further validating tissue specific delivery of the plasmid when a tissue specific Cre is in the right place. In addition, we validated tissue specificity of pFabpl-Cre dependent knockdown of another gene (firefly luciferase gene). **Figure 9H** shows that the *in vivo* transfection and pFabpl-Cre dependent knockdown of the firefly luciferase gene was specific to colorectal tumor tissue, as well as to small and large intestines, but not to kidney or liver. This indicates that the nanoparticles carrying plasmids activate only in specific tissues that are dependent on promoter driven Cre expression. The *in vivo* knockdown of the luciferase gene was strikingly greater in SQ tumors with similarity between colon and intestine tumors (**Figure 9H**), indicating that the transferrin-coated nanoparticles carrying plasmids accumulate into the colon tumors more than into normal intestine/colon cells because of increased expression of transferrin receptor by these tumors as well as by expression of pFabpl-Cre mediated conditional silencing of the firefly luciferase gene (59, 71).

**Figure 10:**
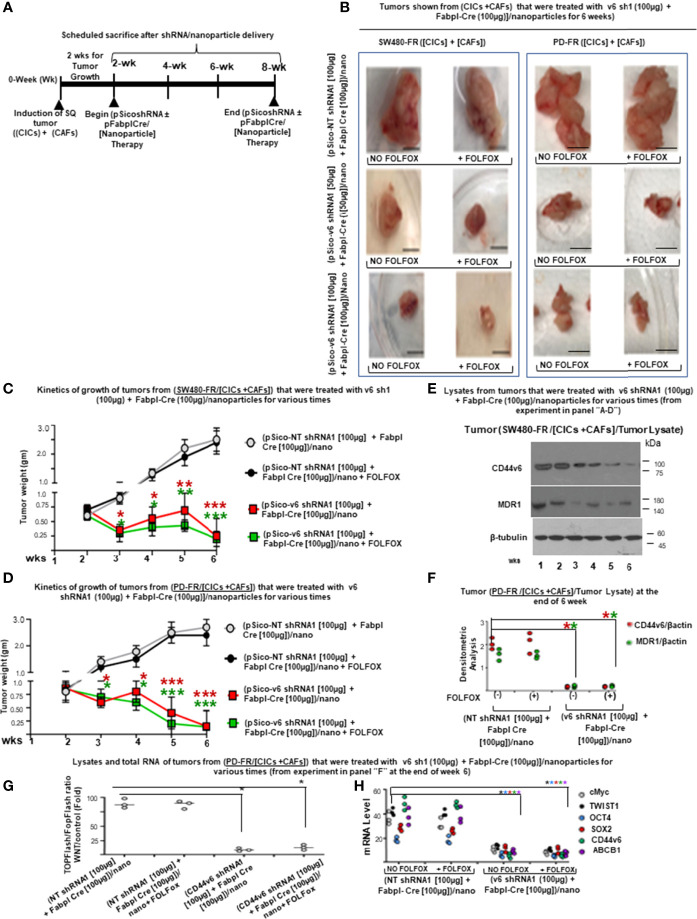
Tissue specific knockdown of CD44v6 by pFabpl-Cre inhibits subcutaneous (SQ) tumor growth of implanted CICs plus CAFs from SW480-FR/SQ tumors and from PD-FR/SQ tumors. **(A)**, Timeline is shown for pSico-v6-shRNA/Nano particle treatment with and without pFabpl-Cre/Nano in xenograft tumors derived from CICs plus CAFs from SW480-FR/SQ tumors and PD-FR/SQ tumors. **(B)**, The dependence of tumors derived from CICs and CAFs on CD44v6 was evaluated in vivo. 2 x 104 FACS sorted CAM(+)CD44v6(+)CD133(+)ALDH1(+) CICs and 6 x 104 CAFs (EpCAM [-]/PDGFR-α were injected into mice. When tumors reached to approximately 0.3 cm3 in volume, treatment was initiated. Detailed treatment procedure is in Methods. Seven immunocompromised mice per group were used. Mice were weighed every other day, and the tumor weights were measured every week for 4 weeks. Representative tumors following sacrifice from three experiments are shown from mice treated as indicated in the schedule of treatment **(A)**. **(C, D)**, Kinetics of relative tumor weights with time are shown during in vivo SQ tumor growth at the indicated weeks that were generated by CICs plus CAFs from SW480-FR/SQ tumors **(C)** and from PD-FR/SQ tumors **(D)** injected (i.p.) with pSico-NT shRNA/Nano, pSico-v6 shRNA/Nano, and (pSico-v6 sh plus pFabpl-Cre)/Nano. Treatments were performed at weeks (wks) 2, 3, 4, and 5 and tumor growth was measured at wks 3, 4, 5, and 6 after treatments. **(E)**, Western blot analyses using CD44v6, MDR1 and β-catenin antibodies in extracts from the various treated xenograft tumors derived from CICs plus CAFs from SW480-FR/SQ tumors collected at 6 weeks from the experiment in **(A–C)** are shown. **(F)**, Densitometric ratios are shown of CD44v6/β -actin and MDR1/β -actin in the Western blot analyses using CD44v6, MDR1 and β−catenin antibodies in extracts from the various treated xenograft tumors derived from CICs plus CAFs and from PD-FR/SQ tumors collected at 6 weeks from the experiment in **(A, B, D)**. The results are means ± SE from four independent experiments (n = 6 mice) **(G)**, QPCR analyses of the indicated stemness related transcription factors from the total RNA from the various treated tumors collected at 6 weeks in **(A, B, D)** are shown. **(H)**, WNT3A/β-catenin reporter activities in the lysates from the tumors collected at 6 weeks from experiments in (A, B, D) are shown. Data **(C, D–H)** are presented as mean ± SD (n = 7); *P < 0.05 compared to appropriate control group. Western blot data (E) are representative of three experiments. Tumor data are presented as mean ± SD (n = 7 mice/group in four independent experiments); **(C–H)**, *P < 0.05. **P < 0.01, ***P were considered significant, Inhibition of tumor growth in (v6 shRNA + pFabpl Cre)/nano, and in (v6 shRNA + pFabpl Cre)/nano plus FOLFOX compared with (NT shRNA + pFabpl Cre)/nano, and in (NT shRNA + pFabpl Cre)/nano plus FOLFOX groups respectively Western blot data are representative of four independent experiments. ANOVA, followed by Bonferroni’s post-hoc test was used to assess the significance in C and D. Student’s t-test was used to assess the significance in **(E–H)**. Scale bars, 1 cm.

We next used the transferrin-nanoparticle delivery of pSicoCD44v6 shRNA plus Fabpl-Cre to determine whether silencing the expression of CD44v6, or of CD44v6-containing variants, can inhibit the tumor cell survival and growth by reducing WNT-CD44v6-MDR1 signaling. The pSicoCD44v6 shRNA1 plus Fabpl-Cre nanoparticles were used *in vivo* to examine the therapeutic potential of targeting CD44v6 in tumors implanted from CICs plus CAFs isolated from a FOLFOX resistant SW480-FR/SQ tumor, and from a patient-derived tumor specimen (PD-FR) (**Figure 10A**, schematic model). Details of the experimental procedures used are in **Figure 10B**. For this experiment, xenograft tumors were established by injecting SW480-FR/CICs in combination with SW480-FR/CAFs, and with PD-FR/CICs in combination with PD-FR/CAFs subcutaneously into immunocompromised mice (schematic model indicated in **Figure 10A**). Cre-mediated conditional silencing of endogenous CD44v6 was used in combination with FOLFOX therapy. Tissue samples from the experiments in **Figure 10B** were used in the follow-up experiments in the **Figures 10B–H**. Administration of Cre-mediated CD44v6shRNA nanoparticles prevented tumor growth (**Figures 10B–D**) and strongly inhibited CD44v6 and MDR1 expressions (**Figure 10E**) in a dose-dependent manner. Moreover, 100 µg/ml plasmid concentration + 1 x FOLFOX had the predominant effect in reducing tumor growth and inhibiting MDR1 expression (**Figures 10B–F**). In agreement with our recent study (55), knocking down CD44v6 reduced stemness related genes including c-Myc, TWIST1, OCT4, SOX2 and ABCB1 (MDR1) substantially in tumors generated from CICs plus CAFs (**Figure 10H**). Moreover, our data in **Figures 10G, H** support our hypothesis that CD44v6-WNT/*β*-catenin signaling regulates the stemness of CICs expressing CD44v6 (**Figure 10H**), and that blocking CD44v6 tissue specifically by conditional silencing has a dramatic effect on reduction of tumor growth (**Figures 10B–D**) by blocking WNT/*β*-catenin specific TOPFlash promoter activity in tumor lysates (**Figure 10G**). Importantly, pSico-v6shRNA1/Nano i.p. administration showed no evidence of toxicity within these experiments or in previous studies using Cre-mediated CD44v6shRNA1 nanoparticles (31, 49). These results provide direct relevance for CD44v6 as a therapeutic target to maintain FOLFOX sensitivity and reduce colon tumor growth by reducing MDR1 expression.

## Discussion

Many studies have shown that CICs may be accountable for tumor resistance to conventional chemotherapy (8, 72). When the resistance is increased through the crosstalk between CICs and the tumor microenvironment, that resistance concept acts through two ways for tumor progression in CICs: intrinsic and extrinsic pathways. The intrinsic mechanisms involve gene mutation, while the extrinsic mechanisms incorporate the production of distinct growth factors and cytokines by the tumor microenvironment leading to the activation of specific signaling pathways (22). This study revealed that paracrine factors secreted by TME cells such as CAFs induce WNT-CD44v6 signaling in CICs to further drive tumor growth in xenograft tumors implanted with CICs combined with CAFs. Interestingly, administration of cytotoxic drugs, including FOLFOX, induces secretion of paracrine factors by CAFs derived from colorectal cancer specimens from patients after cytotoxic drug treatment compared to normal fibroblasts (**Figures 4D–F**).This concept is important because several studies carefully used modified normal fibroblasts in place of CAFs with CRC cell lines to dissect out tumor-stromal interactions (12, 13, 73, 74). Most lethal colorectal tumors are diagnosed at more advanced stages. Thus we used a clinically relevant approach based on CAFs and CICs directly derived from fresh tumors of drug resistant human patient tissues (PD-FUR, or PD-OXAR or PD-FR), or from CAFs and CICs derived from our resistant cell implanted SQ tumors. Using these fresh specimens from patients, we showed a significant increase in CAFs occurrence in cytotoxic drug treated tumors (**Figure 1D**). Interestingly, the CICs isolated from cytotoxic drug treated patient specimens produced very low levels of secretome (secreted factors) (**Supplemental Figure 1A**). Thus, if CAFs truly do evolve from lethal chemotherapy resistant tumors upon drug exposure, then data acquired from modified normal colon fibroblasts may not reflect an accurate picture of the functions of CAFs in supporting CIC growth. Based on data in this study, we postulate that the secretome from CAFs used in our study supports that cytotoxic therapy induced CIC growth/maintenance drives CRC recurrence and mortality.

Most cancers originate from cells that gained cancer-initiating capacity or cancer stemness. These cells are known as CICs that are plastic in nature. Stemness functions of CICs are influenced by extrinsic factors secreted from stroma. Sustained drug resistance and tumorigenic potential of CICs is on TME and especially on the niche-microenvironment where CICs reside. Niches are specialized microenvironments that regulate the fate of CICs by providing cues in the form of both CIC-TME cell contacts and by secreted stromal factors. Here we demonstrate that tumorigenic colorectal cells are included in a rare population of undifferentiated CICs that express CD44v6 (**Figures 2D, E**). Our companion paper (48) revealed that the CIC immunophenotype (CD44v6^high+^) within a tumor is responsible for tumor formation, progression, and resistance to FOLFOX therapies. The interaction between CICs and their tumor niches is strongly linked to the CIC survival/self-renewal (75). Through this tumor-stroma interaction, CICs can preserve the tumor heterogeneity that underlies their important malignant behaviors and therapy resistance (76). Based on this backdrop, our present study revealed that colorectal tumors respond to FOFOX therapy by stimulation of supporting CAFs in the tumor-microenvironment to provide cues in the form of secreted factors (PN and IL17A) to stimulate WNT3A signals to maintain CICs. Our data also suggest that FOLFOX stimulation increased the frequency of CAFs measured by stimulating a relative proportion of fibroblasts to the epithelial component (*α*SMA versus EpCAM). Subsequently, the CAFs create a niche that is chemo resistant by releasing predominantly PN, WNT3A and IL17A. Exogenous addition of either PN, IL17A or WNT3A increased CIC tumorigenic function and maintenance. Especially, these factors were overexpressed by colorectal CAFs in response to FOLFOX with their expression validated directly in patient-derived specimens. Periostin and IL17A sustain a WNT3A-CD44v6 induced CD44v6(+) CIC-maintenance that is shared by WNT ligands. Additionally, our data also revealed that the tumorigenic potential of these CICs together with the CAF subpopulation significantly increased in secondary and tertiary subcutaneous xenograft tumors. In contrast, tumorigenic potential of Non-CICs plus CAFs was completely lost in secondary, tertiary and quaternary subcutaneous xenograft tumors suggesting that Non-CICs are differentiated non-tumorigenic cells. These results provide evidence that drug resistant and long-term tumorigenic potential are restricted to the CD44v6 expressing CIC population, and chemotherapy induces remodeling of the tumor microenvironment to support the tumor cellular hierarchy through secreted factors.

First, using primary cultures derived from the tumors of patients who were treated with cytotoxic regimens (PD-5FUR, PD-OXAR and PD-FR), we showed how FOLFOX induced enrichment of CAFs induces maintenance of CICs for self-renewal capacity as they can be clonally expanded, are exclusively tumorigenic, and are able to differentiate. As PN and IL17A provide important instructive signals from supportive CAFs to stimulate WNT3A, and because CD44v6 regulates these factors in the microenvironment, we focused our attention on these molecules within the CAF’s-secretome. Freshly isolated patient-derived CD44v6+CICs were highly tumorigenic, and as few as 2 x 10^3^ CD44v6+CICs were capable of inducing orthotopic tumor formation in immunocompromised mice. In contrast, as many as 5 x 10^5^ CD44v6 (–) Non-CICs did not result in any tumor formation. Interestingly, when freshly derived CAFs from FOLFOX-resistant tumors were combined with CICs, the resultant tumors attained long term tumorigenic potential with shorter latency, and increased self-renewal capacity as they can be clonally expanded (**Figures 3F–G**).

Second, these factors (PN and IL17A) from CAFs recruit WNT3A ligands and thereby stimulation of WNT-CD44v6 signaling in CICs, which instructs the cellular hierarchy. In our companion paper (48), we showed for the first time that CD44v6 regulated a WNT/*β*-catenin axis, which in turn further transactivates MDR1 and CD44v6 gene expressions in CD44v6(+) CICs. Activation of WNT-CD44v6 signaling has a key role in maintaining the CIC pool in the gut and in promoting self-renewal of colorectal-CICs (64, 77, 78). Stromal elements contribute significantly to maintain the undifferentiated status and the clonogenic activity of the tumorigenic cells (12–14, 79). Our results revealed that this property is not confined only to WNT, but is shared by PN and IL17A through FOLFOX, which augments *β*-catenin activation in colorectal CICs expressing CD44v6 (**Figure 5**).

Third, our results show the direct relevance of PN, WNT3A and IL17A as dominant paracrine signals for the interplay between CICs and CAFs in tumorigenesis. Since CD44v6 regulated WNT3A, as well as IL17A and PN production in CAFs (**Figures 5A, B**), we showed that targeting either WNT3A, or IL17A, or CD44v6 or PN in the CICs + CAFs implanted PD-FR and SW480-FR SQ tumors in immune-deficient mice was effective in reducing colon tumor growth (**Figures 10B–E** versus **Figures 8B–E**). Importantly, these results further indicate that IL17A signaling also has a functional role in CIC-CAF interactions independent of adaptive immune cell functions.

Fifth, we showed that our cell-specific delivery approach (59) to target the CD44v6-induced signaling pathway in colon cancer cells inhibited colon tumor growth (**Figure 10**) significantly compared to the CD44v6 neutralizing antibody mediated reduced tumor growth (**Figure 8**). This cell-specific targeting approach has several advantages and may be easily designed to target other CD44variant-expressing cancer cells, or any cell-surface receptor-expressing cancer cells where the receptor is both a functional marker and a therapeutic target of CICs.

## Conclusion

**Figure 11:**
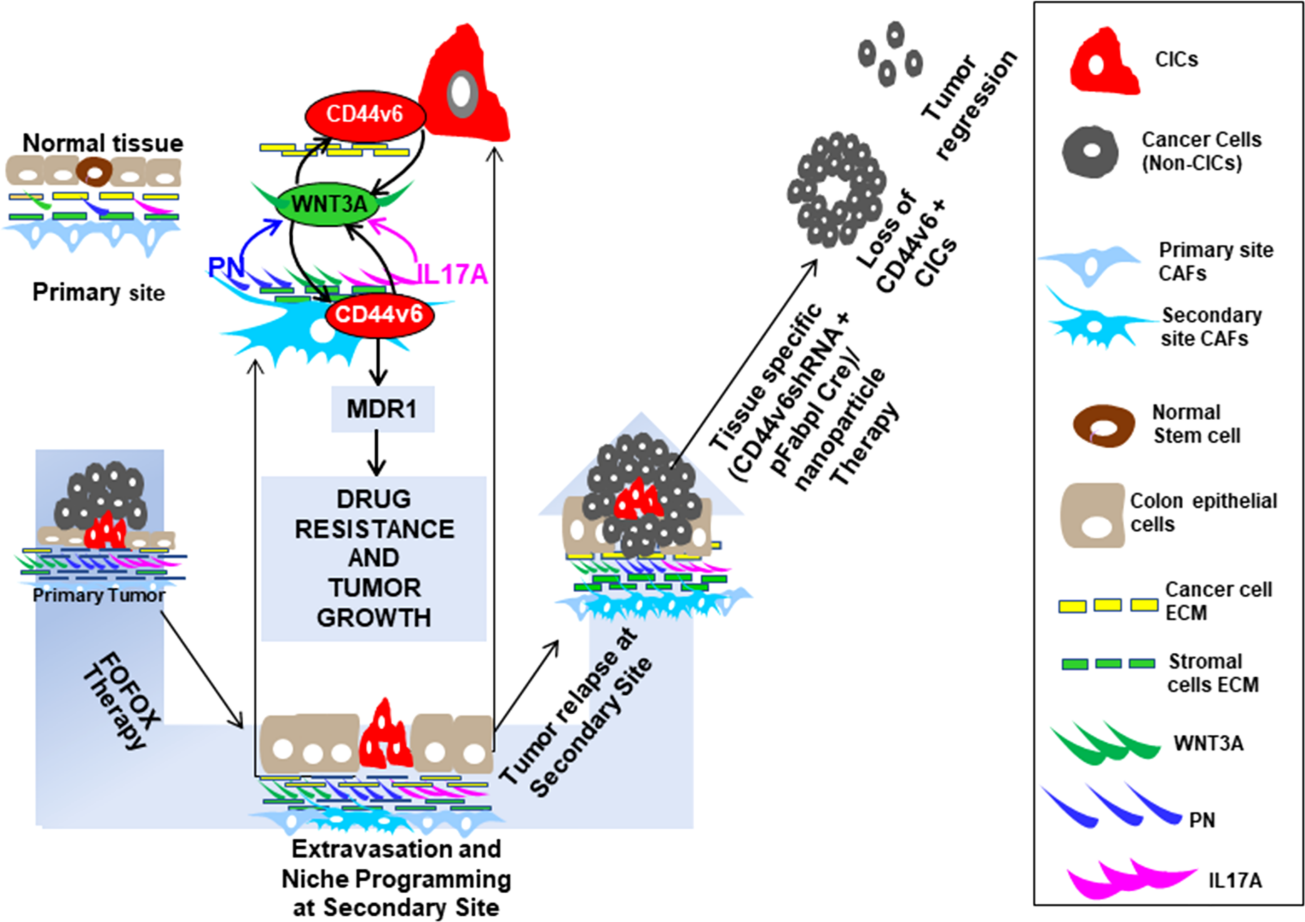
Conclusion Figure. Colon cancer associated fibroblast derived PN and IL17A promote WNT3A induced CD44v6 expression in cancer initiating cells (CICs). CD44v6 positive CICs induce drug resistance and colorectal tumorigenesis by enhancing CD44v6/βcatenin nuclear localization and MDR1 gene expression in CICs. CD44v6 regulated PN, IL17A and WNT3A derived from chemotherapy induced CAFs that contribute to CIC maintenance through induction of the CD44v6 receptor. CD44v6 regulated PN, IL17A and WNT3A mediates the crosstalk between CICs and their niche to permit tumor growth and drug resistance. Importantly, this work identifies that tissue specific knockdown of CD44v6 inhibits functions of CICs and their interaction with CAFs, and thereby significantly suppresses tumor growth.

We found that human colorectal CICs that are defined by CD44v6 expression are exclusively tumorigenic and highly resistant to FOLFOX-therapy. Colorectal-tumor progression is initially triggered by CD44v6(+) CICs. The interactions between CD44v6(+) CICs and their niche are supported by periostin and IL17A (**Figure 11**). Periostin and IL17A simulate WNT3A, which is crucial for augmenting CD44v6(+) CICs that promotes long-lasting tumorigenic potential. It is most intriguing that in an *in vivo* model of orthotopic colorectal cancer, implantation of CAFs with CD44v6(-) Non-CICs virtually abrogated the primary-tumor formation, whereas CAFs with CD44v6(+) CIC populations can reproduce the original tumor in permissive recipients. Since FOLFOX-therapy treated CAFs and CICs express increased levels of CD44v6 that regulates periostin, IL17A and WNT3A, the depletion of CD44v6 from tumors by tissue specific delivery of CD44v6 shRNA almost abrogated the stemness phenotype of CICs with significant inhibition effects on their tumorigenic potential (**Figure 11**). Therefore, we demonstrate that CD44v6 is a CIC marker and a therapeutic target. In summary, the drug resistance ability of colorectal CICs is increased by supported microenvironmental signals that promote the expression of CD44v6, and this indicates that CD44v6 is a molecule that could be clinically exploited both as a biomarker and an effective therapeutic modulator of CRC.

## Data Availability Statement

All the data are included within the manuscript. The names of the repository/repositories and accession number(s) can be found in the article supporting information. Coding nucleotide sequences of the genes were obtained from the NCBI, National Institutes of Health, website (www.ncbi.nlm.nih.gov). Hairpin shRNAs were designed to target a transcript sequence using the Broad Institute GPP Web Portal (http://portals.broadinstitute.org/gpp/public/). Primers were designed by online Primer Quest Tool (https://www.idtdna.com/PrimerQuest/Home/Inde).

## Ethics Statement

The animal study was reviewed and approved by Institutional Review Board (IRB) Statement. The Medical University of South Carolina (MUSC) IRB determined that this research project meets the criteria for “Not Human Subjects”. All animal studies described were approved by Institutional Animal Care and Use Committee (IACUC) (protocol # IACUC – 2019-00829; Approval period 08/03/2020- 09/24/ 20221) at the Medical University of South Carolina (MUSC) and conducted in accordance with the National Institutes of Health Guide for the Care and Use of Animals. Procedures for animal studies were conducted in accordance with the National Institutes of Health Guide for the Care and Use of Animals IACUC-2017-00250 (approval date: 2019/03/14-2021/03/29).

## Author Contributions

The experiments of this work were designed and carried out by SM and SG. The paper was written by SM and SG. VH reviewed, edited the multiple versions of the drafts and final versions of the text, figures, figure legends, participated in designing experiments and supplied reagents. RM edited the drafts, provided reagents, and participated in designing experiments. NK helped in editing the drafts and participated in designing experiments. All authors contributed to the article and approved the submitted version.

## Funding

This work was supported by the: 1) 1R03CA167722-01A1 (to SM and SG); 2) 1K12HL141952-02 (to VH); 3) 2P20GM10399; NIH IDeA Network for SC Biomedical Research Excellence, 4) 02 P30 GM13195901, 5) **19TPA34900016** (to RM); and 6) SCTR grant (UL1 TR001450 for SM). The costs of publication of this article will be defrayed in part by the payment of page charges. This article must therefore be hereby marked “*advertisement*” in accordance with 18 U.S.C. Section 1734 solely to indicate this fact.

## Acknowledgments

The prostate specific pARR2Probasin Cre construct was a gift from Dr. Robert J Matusik, Department of Cell and Developmental Biology, Vanderbilt University, Nashville, TN, USA.

## Conflict of Interest

The authors declare that the research was conducted in the absence of any commercial or financial relationships that could be construed as a potential conflict of interest.

## Publisher’s Note

All claims expressed in this article are solely those of the authors and do not necessarily represent those of their affiliated organizations, or those of the publisher, the editors and the reviewers. Any product that may be evaluated in this article, or claim that may be made by its manufacturer, is not guaranteed or endorsed by the publisher.

## Supplementary Material

Supplementary Figure 1 Exogenous addition of PN and IL17A induces WNT3A secretion and tumor sphere formation in CAFs. **(A)**, Exogenous addition of 20 ng/ml of PN, or IL17A stimulated WNT3A production in CAFs isolated from sensitive and FR tumors of SW480 were assessed by measuring secretion of WNT3A by an ELISA assay. **(B)**, Autocrine expression of two dominant cytokines (IL17A and WNT3A) and of PN were assessed by using ELISA assays on PD-FR CICs (Sphere/CICs) treated with vehicle (DMSO) or with 1 x FOLFOX for 72 hours. **(C)** Percentages of colon tumor sphere formation are shown in CICs of FR-resistant and sensitive cells of SW480 in the absence and presence of 100 ng/ml of either PN-blocking antibody, IL17A-blocking antibody or WNT3A-blocking antibody. Data are presented as Mean ± SD (n = 4 replicates from three independent experiments *, P < 0.05 was considered significant, **(A, B)**, stimulation of secreted protein in treatment groups were compared with vehicle controls; **(C)**, Inhibition of percent of tumor sphere formation in PN, IL17A and WNT3A antibody treated groups compared with IgG control group. Student’s t-test was used to assess the significance.

## Abbreviations

CD44s, Standard isoform of CD44 having no variant exons; CD44v, CD44 splice variant; CD44v6, variant 6 of CD44; PN, periostin; CIC, cancer initiating cell; CAF, cancer initiating fibroblast; CRC, colorectal cancer; 5-FU, 5-fluorouracil; OXA, oxaliplatin; FOLFOX, 5-FU plus OXA plus leucovorin; ALDH1, aldehyde dehydrogenase; LRP6, low-density lipoprotein receptor-related protein; TCF, T-cell factor; LEF, lymphoid enhancer factor; Tf, transferrin; Nano (Tf-PEG-PEI [nanoparticles]); MDR1, multidrug resistance protein 1; IP, immunoprecipitation; WB, Western Blotting; DN, dominant negative; sh, shRNA; SQ, sub cutaneous.
