# Supplementary material for: Interplay Between Chemotherapy-Activated Cancer Associated Fibroblasts and Cancer Initiating Cells Expressing CD44v6 Promotes Colon Cancer Resistance
Source: Front Oncol. 2022 Aug 2;12:906415. doi: 10.3389/fonc.2022.906415 (PMC9380598; doi:10.3389/fonc.2022.906415)
Supplement: Supplementary Figure 1 — Exogenous addition of PN and IL17A induces WNT3A secretion and tumor sphere formation in CAFs. (A), Exogenous addition of 20 ng/ml of PN, or IL17A stimulated WNT3A production in CAFs isolated from sensitive and FR tumors of SW480 were assessed by measuring secretion of WNT3A by an ELISA assay. (B), Autocrine expression of two dominant cytokines (IL17A and WNT3A) and of PN were assessed by using ELISA assays on PD-FR CICs (Sphere/CICs) treated with vehicle (DMSO) or with 1 x FOLFOX for 72 hours. (C) Percentages of colon tumor sphere formation are shown in CICs of FR-resistant and sensitive cells of SW480 in the absence and presence of 100 ng/ml of either PN-blocking antibody, IL17A-blocking antibody or WNT3A-blocking antibody. Data are presented as Mean ± SD (n = 4 replicates from three independent experiments *, P < 0.05 was considered significant, (A, B), stimulation of secreted protein in treatment groups were compared with vehicle controls; (C), Inhibition of percent of tumor sphere formation in PN, IL17A and WNT3A antibody treated groups compared with IgG control group. Student’s t-test was used to assess the significance. [file Presentation_1.pptx]

## Slide 1
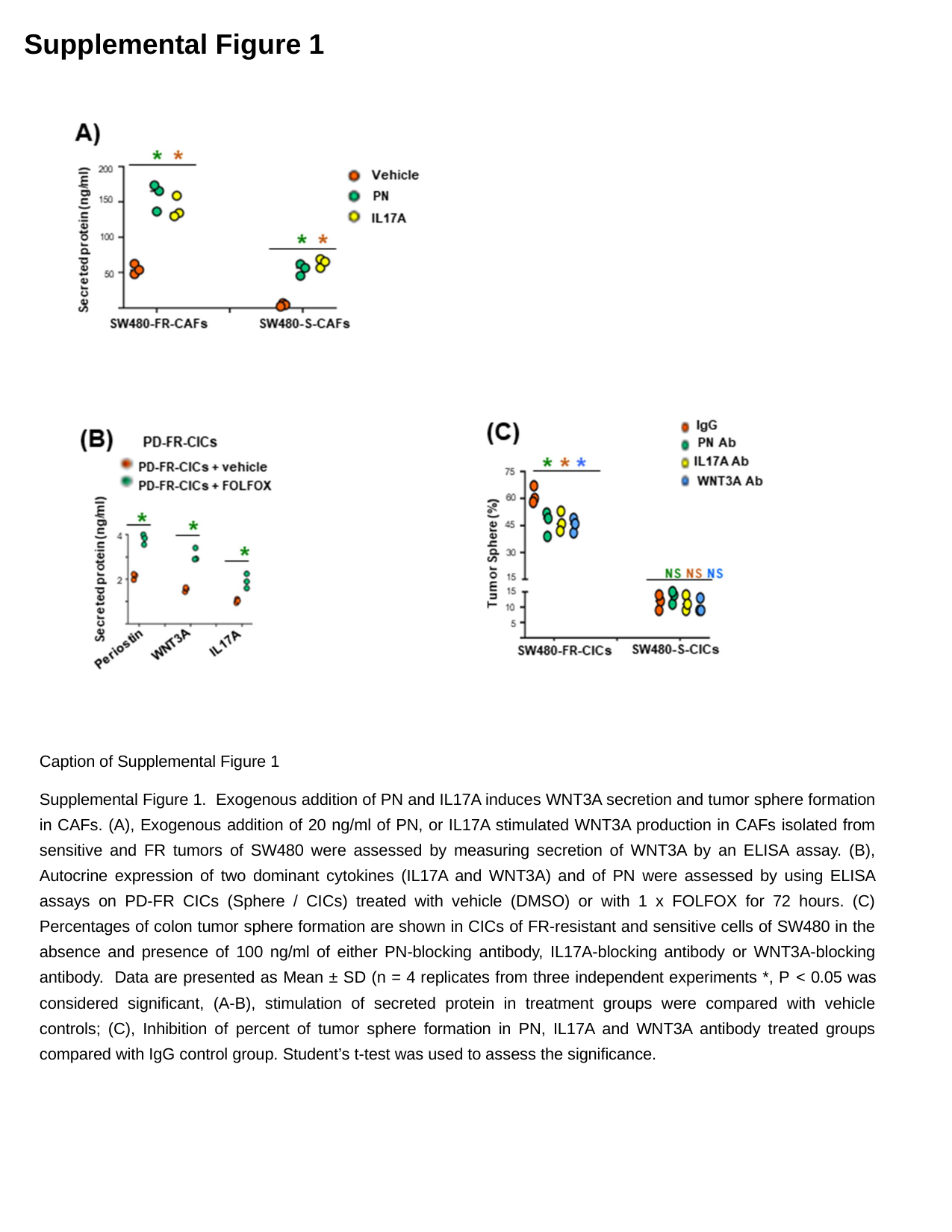

Supplemental Figure 1
Caption of Supplemental Figure 1
Supplemental Figure 1. Exogenous addition of PN and IL17A induces WNT3A secretion and tumor sphere formation in CAFs. (A), Exogenous addition of 20 ng/ml of PN, or IL17A stimulated WNT3A production in CAFs isolated from sensitive and FR tumors of SW480 were assessed by measuring secretion of WNT3A by an ELISA assay. (B), Autocrine expression of two dominant cytokines (IL17A and WNT3A) and of PN were assessed by using ELISA assays on PD-FR CICs (Sphere / CICs) treated with vehicle (DMSO) or with 1 x FOLFOX for 72 hours. (C) Percentages of colon tumor sphere formation are shown in CICs of FR-resistant and sensitive cells of SW480 in the absence and presence of 100 ng/ml of either PN-blocking antibody, IL17A-blocking antibody or WNT3A-blocking antibody. Data are presented as Mean ± SD (n = 4 replicates from three independent experiments *, P < 0.05 was considered significant, (A-B), stimulation of secreted protein in treatment groups were compared with vehicle controls; (C), Inhibition of percent of tumor sphere formation in PN, IL17A and WNT3A antibody treated groups compared with IgG control group. Student’s t-test was used to assess the significance.
